# Parkin targets HIF-1α for ubiquitination and degradation to inhibit breast tumor progression

**DOI:** 10.1038/s41467-017-01947-w

**Published:** 2017-11-28

**Authors:** Juan Liu, Cen Zhang, Yuhan Zhao, Xuetian Yue, Hao Wu, Shan Huang, James Chen, Kyle Tomsky, Haiyang Xie, Christen A. Khella, Michael L. Gatza, Dajing Xia, Jimin Gao, Eileen White, Bruce G. Haffty, Wenwei Hu, Zhaohui Feng

**Affiliations:** 1 Rutgers Cancer Institute of New Jersey, Robert Wood Johnson Medical School, Rutgers, State University of New Jersey, New Brunswick, NJ 08903 USA; 2 Zhejiang Provincial Key Laboratory for Technology & Application of Model Organisms, School of Life Sciences, Wenzhou Medical University, Wenzhou, 325035 China; 3 Division of Hepatobiliary and Pancreatic Surgery, Department of Surgery, First Affiliated Hospital, Zhejiang University School of Medicine, Hangzhou, 310006 China; 4 School of Public Health, Zhejiang University School of Medicine, Hangzhou, 310058 China; 5 Department of Pharmacology, Rutgers, State University of New Jersey, Piscataway, NJ 08854 USA

## Abstract

Mutations in E3 ubiquitin ligase Parkin have been linked to familial Parkinson’s disease. Accumulating evidence suggests that Parkin is a tumor suppressor, but the underlying mechanism is poorly understood. Here we show that Parkin is an E3 ubiquitin ligase for hypoxia-inducible factor 1α (HIF-1α). Parkin interacts with HIF-1α and promotes HIF-1α degradation through ubiquitination, which in turn inhibits metastasis of breast cancer cells. Parkin downregulation in breast cancer cells promotes metastasis, which can be inhibited by targeting HIF-1α with RNA interference or the small-molecule inhibitor YC-1. We further identify lysine 477 (K477) of HIF-1α as a major ubiquitination site for Parkin. K477R HIF-1α mutation and specific cancer-associated Parkin mutations largely abolish the functions of Parkin to ubiquitinate HIF-1α and inhibit cancer metastasis. Importantly, Parkin expression is inversely correlated with HIF-1α expression and metastasis in breast cancer. Our results reveal an important mechanism for Parkin in tumor suppression and HIF-1α regulation.

## Abstract (web summary)

Parkin is an E3 ubiquitin ligase involved in Parkinson’s disease. Parkin has also been linked to cancer suppression but the mechanisms are unclear. Here the authors show that Parkin regulates HIF-1α through ubiquitin-dependent degradation, thus inhibiting metastasis of breast cancer cells.

## Introduction

Mutations in *Parkin* (*PARK2*) cause a familial form of Parkinson’s disease (PD) known as autosomal recessive juvenile PD^1, 2^. Parkin has been reported to play an important role in regulating mitochondrial homeostasis, anti-oxidative stress and mitophagy, which have been linked to the neuroprotective role of Parkin^2, 3^. Like many other RING finger-containing proteins, Parkin can function as an E3 ubiquitin ligase to ubiquitinate and degrade substrate proteins involved in PD such as CDCrel-1, Pael receptor, α-synuclein and synphilin-1^4–8^. Parkin also ubiqutinates protein substrates on the mitochondrial outer membrane following depolarization, triggering mitochondrial elimination by mitophagy^2^. The function of Parkin in controlling turnover of these substrates has been suggested to contribute to its role in prevention of PD^2, 3^.

In addition to its role in PD, accumulating evidence has suggested that Parkin is a tumor suppressor^9–12^. The human *Parkin* gene is localized on chromosome 6q25-27, a region that undergoes frequent loss in cancer. Loss of heterozygosity and copy number loss of *Parkin* have been observed in human cancers, including breast cancer^9, 13^. Parkin expression is frequently downregulated in many types of cancers, including breast cancer^9, 14–16^. Mutations of *Parkin* have been reported in many types of cancers, although its mutation frequency is relatively low^12, 17^. For instance, based on the analysis of data sets from cBioportal (www.cbioportal.org)^18^, *Parkin* is mutated in ~5.6 % of lung squamous cell cancer, 2.4–5.6 % of colorectal cancer, and ~4.6% of gastric cancer^12^. Currently, the mechanism underlying the role of Parkin in tumor suppression is poorly understood and the potential Parkin substrates involved in tumorigenesis remain largely unknown. Cyclin E was reported to be a Parkin substrate^19^, and Parkin was also reported to interact with Cdc20/Cdh1 to mediate ubiquitination and degradation of key mitotic regulators to maintain genomic stability^11^.

Metastasis is the major cause of cancer death. Hypoxia-inducible factor 1 (HIF-1) plays a pivotal role in promoting cancer metastasis^20, 21^. HIF-1 is a heterodimeric transcription factor composed of an oxygen-labile α subunit and a constitutive β subunit. HIF-1 binds to the hypoxia response element (HRE) to regulate gene expression. Under normoxia, HIF-1α is rapidly degraded by tumor suppressor von Hippel-Lindau (VHL) through ubiquitination. Under hypoxia, HIF-1α dissociates from VHL and rapidly accumulates in cells. HIF-1α frequently accumulates in solid tumors, in which hypoxia is a common characteristic^20, 21^. It is important to note that HIF-1α is also regulated by VHL-independent mechanisms in cells. For instance, hypoxia-associated factor (HAF) and SHARP1 can bind to HIF-1α and promote its proteasomal degradation independently of VHL and cellular oxygen tension^22, 23^.

Interestingly, a recent study using quantitative proteomics to systematically analyze the Parkin-dependent ubiquitylome in cells listed HIF-1α as a potential ubiquitination substrate of Parkin^24^. Another recent study reported that Parkin expression reduces HIF-1α levels in glioblastoma cells, however, the mechanism is unknown^25^. These two studies suggest a connection between Parkin and HIF-1α. In this study, we identified HIF-1α as a substrate of Parkin. We found that Parkin binds to HIF-1α and promotes HIF-1α degradation through ubiquitination, which in turn inhibits metastasis of breast cancer cells. Our results reveal an important mechanism underlying the role of Parkin in tumor suppression in cells.

## Results

### HIF-1α is a Parkin-interacting protein

**Fig. 1:**
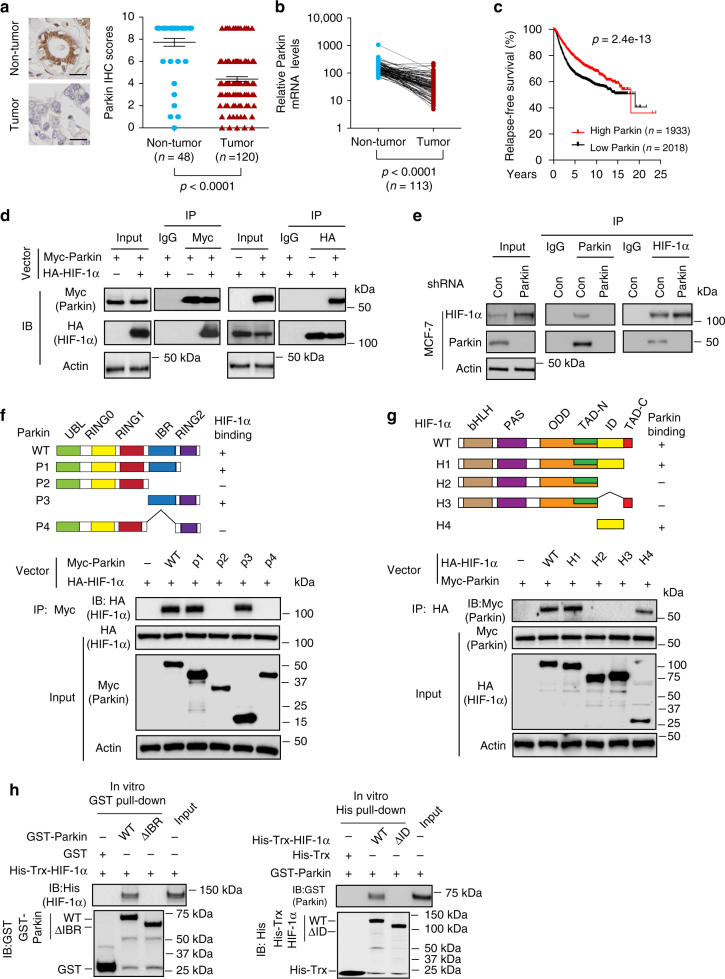
Parkin interacts with HIF-1α. **a** Parkin protein expression was significantly decreased in breast cancer specimens compared with non-tumor breast tissues as analyzed by IHC. Left panel: representative IHC staining images of Parkin in a human breast TMA. Scale bar: 20 μm. Right panel: summary of IHC staining of Parkin in breast cancer specimens (*n* = 120) and non-tumor breast tissues (*n* = 48) in a human breast TMA (TMA-BR2082a; US Biomax). **b** *Parkin* mRNA levels were significantly decreased in human breast cancers compared with matched adjacent non-tumor breast tissues (*n* = 113). The data were obtained from TCGA. In **a**, **b**, *P* < 0.001; two-tailed Student’s *t* test. **c** Low Parkin expression was associated with poor relapse-free survival in breast cancer patients. The data were obtained from Kaplan–Meier plotter. Differences between two survival curves were analyzed using the log-rank (Mantel–Cox) test. **d** Myc-Parkin interacted with HA-HIF-1α in MCF7 cells. Cells were co-transduced with HA-HIF-1α and Myc-Parkin expression vectors for co-IP assays using the anti-Myc (left panels) and anti-HA antibodies (right panels), respectively. **e** Endogenous Parkin interacted with endogenous HIF-1α in MCF7 cells detected by co-IP assays. Endogenous Parkin was knocked down by shRNA in MCF7 cells as a negative control. **f** The IBR domain of Parkin is required for Parkin to interact with HIF-1α. MCF7 cells were transduced with vectors expressing WT or different deletion mutants of Myc-Parkin together with the HA-HIF-1α vector for co-IP assays. **g** The ID domain of HIF-1α is required for HIF-1α to interact with Parkin. MCF7 cells were transduced with vectors expressing WT or different deletion mutants of HA-HIF-1α vectors together with the Myc-Parkin vector for co-IP assays. **h** The interaction between Parkin and HIF-1α analyzed by in vitro GST (left) and His (right) pull-down assays, respectively, using purified GST-Parkin and His-Trx-HIF-1α proteins

Parkin expression has been reported to be frequently decreased in cancer, including breast cancer^9, 14, 16^. Consistently, we found that Parkin protein levels were significantly lower in breast cancer samples (*n* = 120) compared with non-tumor breast tissues (*n* = 48) in a tissue microarray (TMA; TMA-BR2082a from US Biomax) as examined by immunohistochemistry (IHC) staining (Fig. 1a). Analysis of breast cancer data sets from The Cancer Genome Atlas (TCGA) showed that *Parkin* mRNA levels were significantly decreased in breast cancer specimens compared with paired adjacent non-tumor tissues (*n* = 113; Fig. 1b). The Parkin downregulation was not linked to any specific breast tumor subtype in terms of ER, PR or HER2 status (Supplementary Fig. 1a). Compared with normal breast tissues and normal breast MCF10A cells, Parkin expression was decreased in various breast cancer cells, including MCF7 (ER + /PR + /HER2−), MDA-MB231 (ER−/PR−/HER2−), T47D (ER + /PR + /HER2−), SK-BR3 (ER−/PR−/HER2+), and ZR-75-1 (ER + /PR + /HER2+) (Supplementary Fig. 1b). On the basis of the data sets from cBioportal^18, 26^, *Parkin* mutations are observed in < 1% of breast cancer, suggesting that mutation is not a major mechanism contributing to the frequent downregulation of Parkin in breast cancer (Supplementary Fig. 1c). Results from Kaplan–Meier plotter, an online survival analysis tool (http://kmplot.com)^27^, showed that low *Parkin* mRNA expression in breast cancer was significantly associated with poor prognosis of patients (*n* = 3951; Fig. 1c). These results suggest an important role of Parkin in breast cancer.

To further investigate the mechanism underlying the role of Parkin in tumor suppression, we searched for potential ubiquitination substrates of Parkin by screening for potential Parkin-interacting proteins using two sequential rounds of immunoprecipitation (IP) followed by liquid chromatography-tandem mass spectrometry (LC-MS/MS) assays in MCF7 cells transduced with a pLPCX-Myc-Parkin vector to express Myc-Parkin or with the empty vector as a control. HIF-1α was identified as a potential Parkin-interacting protein by LC-MS/MS analysis (Supplementary Table 1). Several known Parkin-interacting proteins, including HSP70, Rpn10, 14-3-3 and Tubulin^28–30^, were also among the list of potential Parkin-interacting proteins identified by the LC-MS/MS assays, validating our approach (Supplementary Table 1). The interaction between Parkin and HIF-1α was confirmed by co-IP followed by western-blot assays in MCF7 cells ectopically expressing Myc-Parkin and HA-HIF-1α. As shown in Fig. 1d, HA-HIF-1α was co-precipitated by the anti-Myc antibody, and Myc-Parkin was co-precipitated by the anti-HA antibody, indicating that Myc-Parkin interacted with HA-HIF-1α in cells. The interaction between endogenous Parkin and HIF-1α was also observed in MCF7 cells (Fig. 1e), T47D and MCF10A cells (Supplementary Fig. 2) by co-IP followed by western-blot assays.

To identify the domain of Parkin that interacts with HIF-1α, wild-type (WT) and different Myc-tagged deletion mutants of Parkin were constructed and co-transfected with vectors expressing HA-HIF-1α into MCF7 cells for co-IP assays. The results in Fig. 1f showed that the IBR (In-between RING) domain of Parkin is required for Parkin to interact with HIF-1α. We further mapped the domain of HIF-1α that interacts with Parkin by co-transfecting Myc-Parkin vectors and vectors expressing different HA-tagged deletion mutants of HIF-1α into MCF7 cells using co-IP assays. As shown in Fig. 1g, the ID domain (Inhibitory domain) of HIF-1α is required for HIF-1α to interact with Parkin.

To further investigate whether Parkin interacts with HIF-1α, in vitro GST and His pull-down assays were performed using GST-Parkin and His-Trx-HIF-1α proteins purified from bacteria. The results from in vitro GST pull-down assays showed that GST-Parkin interacted with His-HIF-1α in vitro, which was disrupted by deletion of the IBR domain of Parkin (ΔIBR Parkin) (Fig. 1h; left panels). Results from in vitro His pull-down assays showed that His-Trx-HIF-1α interacted with GST-Parkin in vitro, which was disrupted by deletion of the ID domain of HIF-1α (ΔID HIF-1α) (Fig. 1h; right panels). Furthermore, the GST tag did not interact with His-Trx-HIF-1α (Fig. 1h; left panels), and the His-Trx tag did not interact with GST-Parkin (Fig. 1h; right panels). Taken together, these results indicate that Parkin interacts with HIF-1α.

### Parkin downregulates HIF-1α protein levels in cells

**Fig. 2:**
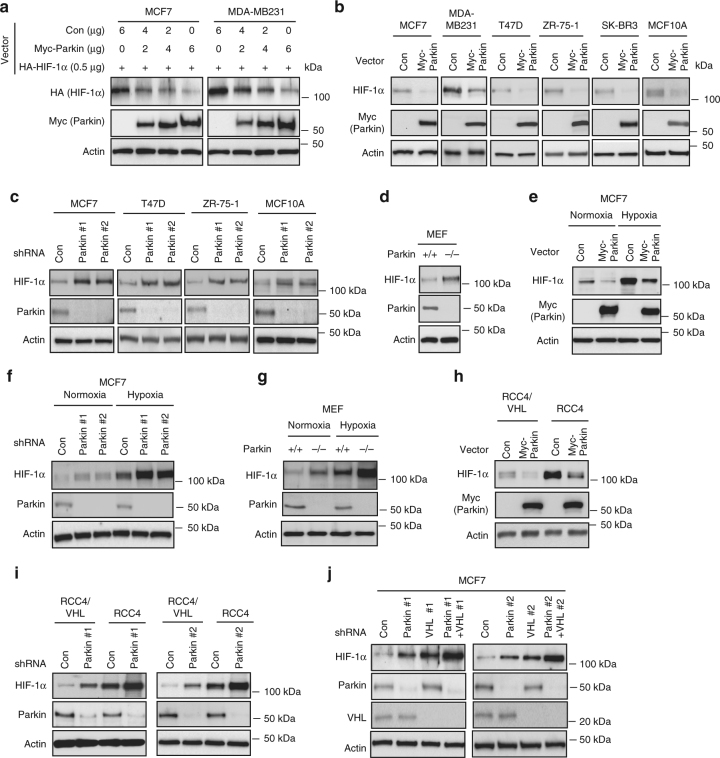
Parkin negatively regulates HIF-1α protein levels in cells. **a** Myc-Parkin expression reduced levels of exogenous HA-HIF-1α protein in MCF7 and MDA-MB231 cells. Cells were transfected with the HA-HIF-1α vector together with varying amounts of Myc-Parkin or empty control (Con) vectors. **b** Ectopic Myc-Parkin expression reduced levels of endogenous HIF-1α protein in different human breast cells. **c** Knockdown of endogenous Parkin by 2 different shRNA vectors increased levels of endogenous HIF-1α protein in different human breast cells. **d** HIF-1α protein levels were higher in *Parkin*−/− MEFs compared with *Parkin* + / + MEFs. **e** Myc-Parkin expression downregulated HIF-1α protein levels in MCF7 cells under both normoxic (20% O_2_) and hypoxic (1% O_2_) conditions. **f** Knockdown of Parkin increased HIF-1α protein levels in MCF7 cells under both normoxic and hypoxic conditions. **g** Higher HIF-1α protein levels in *Parkin*−*/*− MEFs than *Parkin + / + *MEFs under both normoxic and hypoxic conditions. **h** Myc-Parkin expression reduced the levels of endogenous HIF-1α protein in both VHL-deficient RCC4 cells and VHL-proficient RCC4/VHL cells. **i** Knockdown of Parkin increased HIF-1α protein levels in both RCC4 and RCC4/VHL cells. **j** Knockdown of Parkin in MCF7 cells with VHL knockdown further increased HIF-1α protein levels

To investigate whether Parkin regulates HIF-1α protein levels in cells, MCF7 and MDA-MB231 cells were co-transduced with vectors expressing Myc-Parkin and HA-HIF-1α, respectively. Ectopic Myc-Parkin expression reduced HA-HIF-1α protein levels in both cells in a dose-dependent manner (Fig. 2a). Similarly, ectopic Myc-Parkin downregulated endogenous HIF-1α levels in different breast cancer cells and MCF10A cells (Fig. 2b). We further examined whether endogenous Parkin negatively regulates HIF-1α in cells by knocking down endogenous Parkin using 2 different shRNA vectors in the aforementioned different breast cells (excluding MDA-MB231 and SK-BR3 cells, which express low levels of Parkin as shown in Supplementary Fig. 1b). Parkin knockdown increased endogenous HIF-1α protein levels in cells (Fig. 2c). HIF-1α protein levels were further examined in *Parkin + / + *and *Parkin*−*/*− mouse embryonic fibroblasts (MEFs), which are widely used in the study of Parkin^11, 31, 32^. Higher HIF-1α levels were observed in *Parkin*−*/*− MEFs than in *Parkin + / + *MEFs (Fig. 2d).

Parkin was reported to regulate mRNA expression of specific genes^33, 34^. To investigate whether Parkin downregulates HIF-1α protein through repression of *HIF-1α* mRNA in cells, *HIF-1α* mRNA levels were examined in breast cells. Neither ectopic Myc-Parkin expression nor knockdown of endogenous Parkin affected *HIF-1α* mRNA levels as analyzed by quantitative Taqman real-time PCR (Supplementary Fig. 3a–c). Together, these results indicate that Parkin negatively regulates HIF-1α at the protein level in cells.

VHL plays an important role in HIF-1α regulation. Under normoxic conditions, HIF-1α is rapidly degraded by VHL in cells^20, 21^. Our results showed that the effect of Parkin on HIF-1α was independent of oxygen levels unlike VHL (Fig. 2e–g). Myc-Parkin expression decreased HIF-1α protein levels in MCF7 cells under both normoxic and hypoxic conditions (Fig. 2e), whereas Parkin knockdown increased HIF-1α levels in MCF7 cells under both normoxic and hypoxic conditions (Fig. 2f). Furthermore, higher HIF-1α protein levels were observed in *Parkin*−/− MEFs than *Parkin* + / + MEFs under both normoxic and hypoxic conditions (Fig. 2g).

To test whether the downregulation of HIF-1α by Parkin is VHL-dependent, VHL-deficient renal cell carcinoma cells RCC4 and their isogenic cells with stable ectopic expression of VHL (RCC4/VHL) were used to investigate the effect of Parkin on HIF-1α^35^. Myc-Parkin expression reduced HIF-1α protein levels in both RCC4 and RCC4/VHL cells (Fig. 2h), while knockdown of Parkin increased HIF-1α protein levels in both cells (Fig. 2i). Similar results were observed in MCF7 cells; while knockdown of VHL increased HIF-1α protein levels in MCF7 cells, knockdown of Parkin in MCF7 cells with VHL knockdown further increased HIF-1α protein levels (Fig. 2j). These results suggest that Parkin downregulates HIF-1α protein levels in a VHL-independent manner.

### Parkin negatively regulates HIF-1-α transcriptional activity

**Fig. 3:**
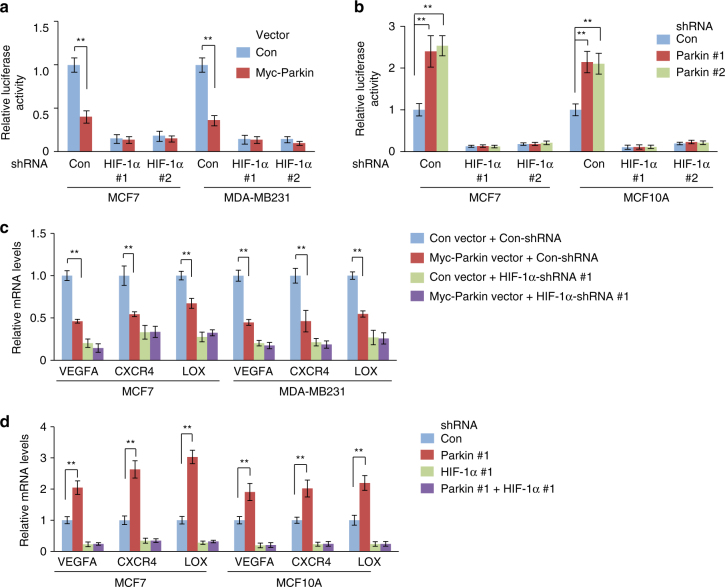
Parkin negatively regulates HIF-1α transcriptional activity. **a** Myc-Parkin expression inhibited HIF-1α luciferase reporter activities in cells. **b** Parkin knockdown increased HIF-1α luciferase reporter activities in cells. **c** Myc-Parkin expression reduced mRNA expression of HIF-1α target genes in cells. **d** Parkin knockdown increased mRNA expression of HIF-1α target genes in cells. Gene expression was measured by quantitative Taqman real-time PCR and normalized with *actin*. In **c**, HIF-1α was knocked down by 2 different shRNA vectors, and in **d**, HIF-1α and Parkin were knocked down by 2 different shRNA vectors. Similar results were observed for 2 different HIF-1α and Parkin shRNA vectors, and only results from one shRNA vector were presented for the sake of clarity. The data present mean ± S.D. (*n* = 6). **P < 0.001; two-tailed Student's *t* test

As a critical subunit of the HIF-1 transcription factor complex, HIF-1α binds to HREs in its target genes to regulate their expression^20, 21^. Here we examined whether Parkin affects the transcriptional activity of HIF-1α. First, luciferase reporter assays were performed using a HIF-1α luciferase reporter vector containing the promoter region of *VEGFA*, a well-known HIF-1α target gene^36^. Expression of Myc-Parkin significantly reduced HIF-1α luciferase reporter activities in both MCF7 and MD-MB231 cells (Fig. 3a), whereas knockdown of Parkin significantly increased HIF-1α luciferase reporter activities in MCF7 and MCF10A cells that have relatively higher endogenous Parkin levels (Fig. 3b). Similar results were also observed in T47D breast cancer cells (Supplementary Fig. 4a, b). Furthermore, Parkin inhibited the HIF-1α luciferase reporter activities under both normoxic and hypoxic conditions (Supplementary Fig. 4c, d). To confirm that the inhibitory effect of Parkin on luciferase activity of the reporter vector is HIF-1α-dependent, HIF-1α was knocked down by 2 different shRNA vectors. HIF-1α knockdown dramatically decreased luciferase activities of the reporter vector. Notably, the inhibitory effect of Myc-Parkin on luciferase activity observed in cells with HIF-1α knockdown was much less pronounced compared with control cells (Fig. 3a; Supplementary Fig. 4a). Furthermore, HIF-1α knockdown largely abolished the promoting effect of Parkin knockdown on the HIF-1α luciferase reporter activity (Fig. 3b and Supplementary Fig. 4b). These results indicate that Parkin inhibits HIF-1α transcriptional activity.

To confirm that Parkin inhibits HIF-1α transcription activity, we examined the mRNA levels of several well-known HIF-1α target genes, including *VEGFA, CXCR4* and *LOX* ^20, 21^, using Taqman real-time PCR in cells with ectopic Myc-Parkin expression or Parkin knockdown. Myc-Parkin expression reduced mRNA levels of these genes in MCF7 and MDA-MB231 cells (Fig. 3c), whereas Parkin knockdown increased their mRNA levels in MCF7 and MCF10A cells, which have relatively higher endogenous Parkin levels (Fig. 3d). Similar results were also observed in T47D breast cancer cells (Supplementary Fig. 4e, f). HIF-1α knockdown significantly decreased mRNA levels of these genes, indicating that these genes are regulated by HIF-1α in these cells (Fig. 3c, d; Supplementary Fig. 4e, f). Notably, compared with cells transduced with control shRNA vectors, cells with HIF-1α knockdown exhibited a much less pronounced inhibitory effect of Myc-Parkin on these genes, suggesting that Parkin represses the expression of these genes through downregulation of HIF-1α (Fig. 3c; Supplementary Fig. 4e). Furthermore, HIF-1α knockdown largely abolished the promoting effect of Parkin knockdown on the mRNA levels of these genes (Fig. 3d; Supplementary Fig. 4f). Collectively, these results indicate that Parkin inhibits HIF-1α transcriptional activity.

### Parkin promotes ubiquitination and degradation of HIF-1α

**Fig. 4:**
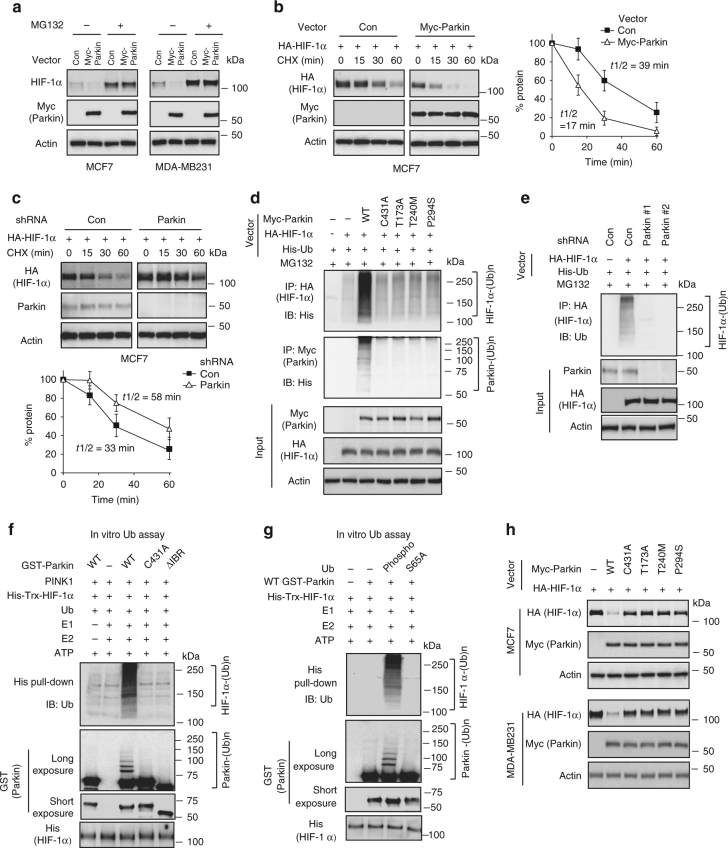
Parkin promotes HIF-1α protein degradation through ubiquitination. **a** Proteasome inhibitor MG132 inhibited the downregulation of HIF-1α protein levels induced by Myc-Parkin expression in MCF7 and MDA-MB231 cells. **b** Myc-Parkin expression decreased HIF-1α protein half-life in cells. MCF7 cells with ectopic Myc-Parkin expression and control cells were transfected with the HA-HIF-1α vector. The cells were treated with 50 µg ml^−1^ CHX for indicated time periods before being collected for western-blot assays. **c** Knockdown of endogenous Parkin increased HIF-1α protein half-life in MCF7 cells. In **b**, **c**, the data present mean ± S.D. (*n* = 3). **d** The effects of expression of Myc-Parkin and its mutants on ubiquitination of HA-HIF-1α and autoubiquitination of Parkin in MCF7 cells analyzed by in vivo ubiquitination assays. **e** Knockdown of endogenous Parkin decreased the ubiquitination of HA-HIF-1α in MCF7 cells analyzed by in vivo ubiquitination assays. **f** Parkin promoted HIF-1α ubiquitination as detected by in vitro ubiquitination assays performed by incubating purified GST-Parkin and His-Trx-HIF-1α proteins in the presence of recombinant E1, E2, ubiquitin (Ub) and PINK1 proteins in vitro. **g** S65A Ub largely abolished ubiquitination of HIF-1α by Parkin in vitro. In vitro ubiquitination assays performed by incubating purified GST-Parkin and His-Trx-HIF-1α proteins in the presence of phosphorylated (Phospho) Ub or S65A Ub. **h** Mutations of Parkin that impaired Parkin’s ubiquitination activity impaired the ability of Myc-Parkin to degrade HA-HIF-1α protein in MCF7 and MDA-MB231 cells

To investigate whether Parkin negatively regulates HIF-1α through ubiquitin-proteasome degradation, MCF7 and MDA-MB231 cells transduced with the Myc-Parkin vector were treated with the proteasome inhibitor MG132, and HIF-1α protein levels were analyzed. MG132 treatment largely abolished the inhibitory effect of Myc-Parkin on HIF-1α protein levels in cells (Fig. 4a). To investigate whether Parkin affects protein stability of HIF-1α, the protein half-life of HIF-1α was analyzed. MCF7 cells with Myc-Parkin expression and their control cells were transduced with the HA-HIF-1α expression vector before they were treated with protein synthesis inhibitor cyclohexamide (CHX) for different time periods. Compared with control cells transduced with the empty vector, cells transduced with the Myc-Parkin vector exhibited reduced half-life of HA-HIF-1α protein (Fig. 4b). Importantly, knockdown of endogenous Parkin increased the half-life of HA-HIF-1α protein in MCF7 cells (Fig. 4c).

To investigate whether Parkin promotes HIF-1α degradation through ubiquitination, in vivo ubiquitination assays were employed. It has been reported that the conserved cysteine C431 of Parkin is required for ubiquitin ligase activity of Parkin and C431A mutation compromises ubiquitin ligase activity of Parkin^37, 38^. T173A, T240M and P294S, cancer-associated mutations of *Parkin*, have been reported to impair ubiquitin ligase activity of Parkin, which in turn abrogates the tumor suppressive function of Parkin^39^. These four mutants were constructed and used for in vivo ubiquitination assays. MCF7 cells were co-transfected with WT or mutant Myc-Parkin vectors together with vectors expressing HA-HIF-1α and His-ubiquitin (His-Ub), respectively. Cells with Myc-Parkin expression displayed increased ubiquitination of HA-HIF-1α compared with cells transfected with the control vector (Fig. 4d). Notably, these four mutations markedly reduced the ability of Parkin to ubiquitinate HA-HIF-1α in MCF7 cells. Parkin can ubiquitinate itself (autoubiquitination), which has been used to reflect ubiquitin ligase activity of Parkin^40, 41^. As shown in Fig. 4d, WT Parkin displayed clear autoubiquitination, and all of these four Parkin mutations impaired Parkin autoubiquitination. Furthermore, knockdown of endogenous Parkin decreased HA-HIF-1α ubiquitination in MCF7 cells (Fig. 4e). CDCrel-1 is a well-known substrate of Parkin, which can be ubiquitinated and degraded by Parkin^4, 42^. Our results showed that ectopic expression of WT but not C341A mutant Myc-Parkin markedly promoted ubiquitination and degradation of HA-CDCrel-1 in MCF7 cells (Supplementary Fig. 5a, b), which validated our experimental systems.

The ubiquitination of HIF-1α by Parkin was confirmed by in vitro ubiquitination assays using purified GST-Parkin and His-Trx-HIF-1α proteins. WT GST-Parkin promoted His-Trx-HIF-1α ubiquitination and displayed GST-Parkin autoubiquitination in the presence of recombinant E1, E2, ubiquitin and PINK1 proteins in vitro (Fig. 4f). The C431A mutation markedly reduced GST-Parkin autoubiquitination and His-Trx-HIF-1α ubiquitination in vitro (Fig. 4f). The IBR domain of Parkin was shown to be required for the Parkin–HIF-1α interaction (Fig. 1h), and was recently reported to be important for ubiquitin ligase activity of Parkin^43^. Deletion of the IBR domain of Parkin (ΔIBR) largely abolished His-Trx-HIF-1α ubiquitination and Parkin autoubiquitination in vitro (Fig. 4f). Our results also showed that WT but not the C431A GST-Parkin protein led to ubiquitination of His-Trx-CDCrel-1 protein in vitro (Supplementary Fig. 5c), which validated our in vitro experimental systems. PINK1 was added to the in vitro ubiquitination reactions since PINK1 has been reported to activate Parkin through phosphorylation of the ubiquitin-like (UBL) domain of Parkin and ubiquitin as well, which is required for Parkin activity^44–46^. Consistent with these reports^44–46^, our results from in vivo ubiquitination assays showed that knockdown of endogenous PINK1 by 2 different siRNA oligos reduced the ubiquitination of HIF-1α by Parkin in MCF7 cells (Supplementary Fig. 6). Our results from in vitro ubiquitination assays further showed that while GST-Parkin ubiquitinated His-Trx-HIF-1α in the presence of phosphorylated ubiquitin, phosphorylation-deficient ubiquitin S65A, a mutant that cannot be phosphorylated by PINK1^46^, largely abolished the function of Parkin to ubiquitinate HIF-1α (Fig. 4g).

We further examined whether above-mentioned four Parkin mutants can impair Parkin’s ability to degrade HIF-1α in cells. As shown in Fig. 4h, these four mutations largely abolished the ability of Parkin to degrade HA-HIF-1α in MCF7 and MDA-MB231 cells. Taken together, these results indicate that Parkin downregulates HIF-1α through ubiquitination and proteasomal degradation.

### Ubiquitination of HIF-1α at lysine 477 by Parkin

**Fig. 5:**
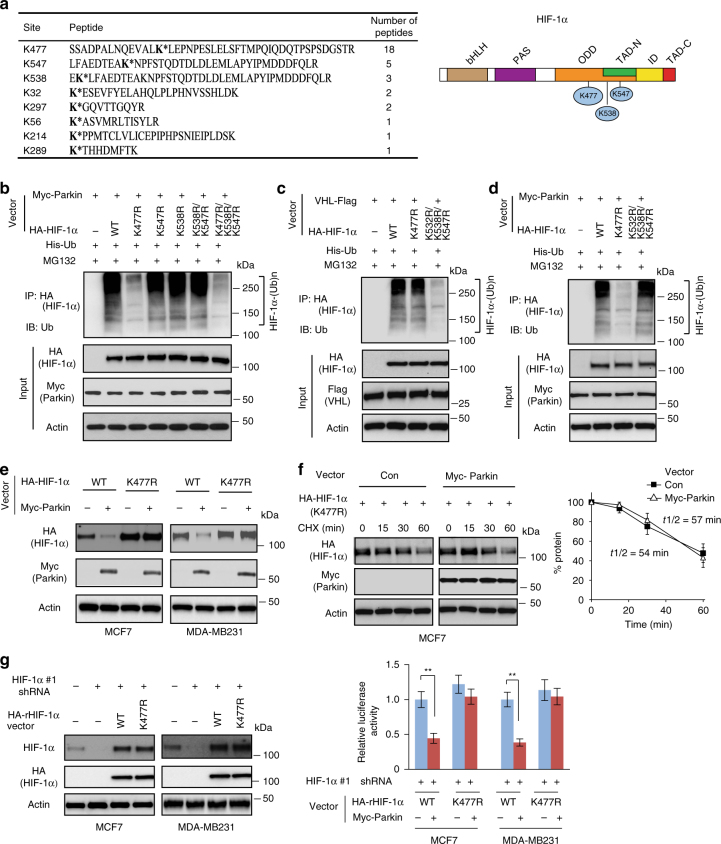
Ubquitination of HIF-1α by Parkin at lysine 477. **a** Left panel: sequences and counts of peptides containing potential ubiquitination sites identified by LC-MS/MS analysis in MCF7 cells with ectopic expression of HA-HIF-1α, Myc-Parkin and His-Ub. *: represents potential lysine ubiquitination site. Right panel: Positions of the top three potential ubiquitination sites in HIF-1α, including K477, K538 and K547. **b** K477 mutation (K477R) largely abolished ubiquitination of HIF-1α by Parkin. MCF7 cells were transfected with indicated vectors for in vivo ubiquitination assays. **c** VHL efficiently ubiquitinated K477R but not K532R/K538R/K547R mutant HA-HIF-1α in MCF7 cells analyzed by in vivo ubiquitination assays. **d** Parkin efficiently ubiquitinated K532R/K538R/K547R but not K477R mutant HA-HIF-1α in MCF7 cells analyzed by in vivo ubiquitination assays. **e** K477R mutation largely abolished the negative regulation of HA-HIF-1α protein by Myc-Parkin in MCF7 and MDA-MB231 cells. Cells were transduced with vectors expressing WT or K477R HA-HIF-1α together with Myc-Parkin vectors. **f** Myc-Parkin expression did not affect the K477R HA-HIF-1α protein half-life. MCF7 cells with ectopic Myc-Parkin expression and control cells were transfected with K477R HA-HIF-1α. The data present mean ± S.D. (*n* = 3). **g** K477R mutation largely abolished the inhibitory effect of Parkin on HIF-1α luciferase reporter activities in cells. MCF7 and MDA-MB231 cells were transduced with HIF-1α-shRNA #1 to knock down endogenous HIF-1α, and then transduced with vectors expressing WT or K477R HA-HIF-1α resistant to HIF-1α-shRNA (HA-rHIF-1α) (left panels). Cells were then transfected with the HIF-1α luciferase reporter vector together with Myc-Parkin vectors for luciferase reporter assays (right panel). The data present mean ± S.D. (*n* = 6). **: *P* < 0.001; two-tailed Student's *t* test

To identify the ubiquitination sites of HIF-1α for Parkin, LC-MS/MS analysis was performed using HA-HIF-1α immunoprecipitated from MCF7 cells co-transfected with vectors expressing Myc-Parkin, HA-HIF-1α and His-Ub, respectively. K477, K547 and K538 were identified as the top three putative ubiquitination sites for Parkin (Fig. 5a). To investigate whether these three sites are major ubiquitination sites for Parkin, different vectors expressing HA-HIF-1α containing single (K477R, K547R, or K538R), double (K538R/K547R) or triple (K477R/K538R/K547R) mutations were constructed. The results from in vivo ubiquitination assays in MCF7 cells showed that compared with WT HA-HIF-1α, K477R mutation largely abolished the ability of Myc-Parkin to ubiquitinate HA-HIF-1α, whereas K547R, K538R, or K538R/K547R mutations did not clearly reduce the ability of Myc-Parkin to ubiquitinate HA-HIF-1α. Compared with K477R mutation, K477R/K538R/K547R mutations did not further inhibit the ubiquitination of HA-HIF-1α by Parkin, suggesting that K477 of HA-HIF-1α is the major ubiquitination site for Parkin (Fig. 5b).

HIF-1α was reported to be ubiquitinated by VHL at K532, K538 and K547^47, 48^. Our results from in vivo ubiquitination assays in MCF7 cells showed that VHL efficiently ubiquitinated both WT and K477R HA-HIF-1α but not K532R/K538R/K547R mutant HA-HIF-1α (Fig. 5c). In contrast, Parkin efficiently ubiquitinated K532R/K538R/K547R HA-HIF-1α, indicating that Parkin and VHL ubiquitinate HIF-1α at different sites (Fig. 5d). Furthermore, K477R mutation did not affect the interaction between Myc-Parkin and HA-HIF-1α in MCF7 cells, indicating that the inability of Myc-Parkin to ubiquitinate K477R HA-HIF-1α is not due to the disruption of Parkin–HIF-1α interaction caused by K477R mutation (Supplementary Fig. 7).

Consistent with its effect on HA-HIF-1α ubiquitination, K477R mutation largely abolished the promoting effect of Myc-Parkin on HA-HIF-1α degradation in both MCF7 and MDA-MB231 cells (Fig. 5e). Compared with WT HA-HIF-1α (Fig. 4b), K477R mutant HA-HIF-1α displayed a longer protein half-life (Fig. 5f). Furthermore, Myc-Parkin expression reduced the half-life of WT HA-HIF-1α (Fig. 4b), but not the half-life of K477R HA-HIF-1α in MCF7 cells (Fig. 5f).

We further investigated whether K477R mutation can largely abolish the inhibitory effect of Parkin on HIF-1α luciferase reporter activity in cells. MCF7 and MDA-MB231 cells were transduced with shRNA vectors to knock down endogenous HIF-1α, and then transduced with vectors expressing WT or K477R HA-HIF-1α resistant to HIF-1α shRNA (HA-rHIF-1α) (Fig. 5g; left panels). These cells were then used for HIF-1α luciferase reporter assays. K477R mutation largely abolished the inhibitory effect of Parkin on HIF-1α luciferase reporter activity in cells (Fig. 5g; right panel). Collectively, these results indicate that K477 of HIF-1α is a major ubiquitination site for Parkin.

### Parkin inhibits cell migration and invasion through HIF-1α

**Fig. 6:**
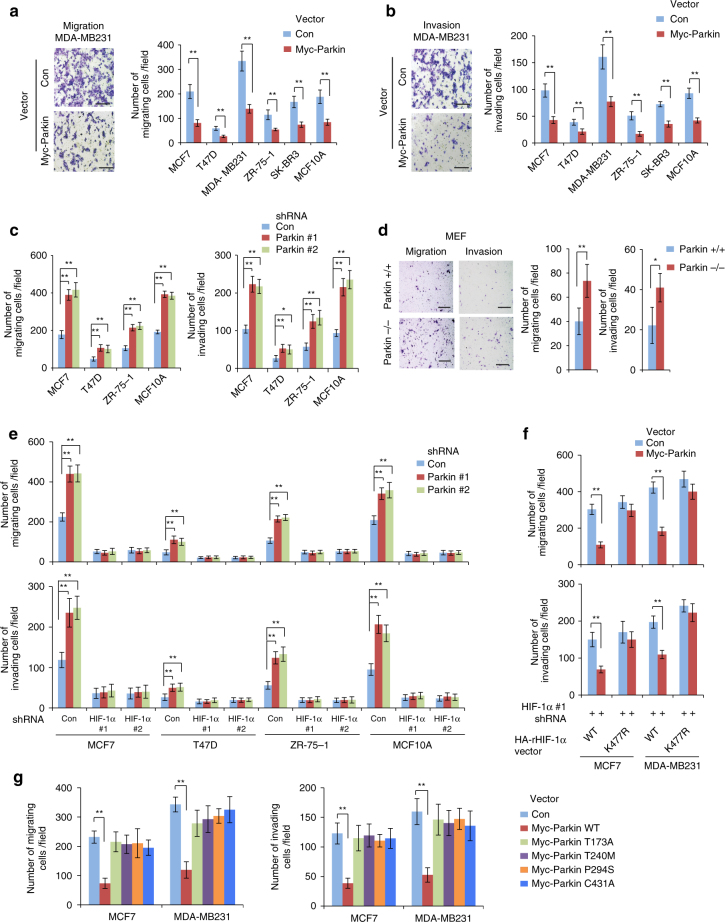
Parkin inhibits migration and invasion of human breast cancer cells through negative regulation of HIF-1α. **a**, **b** Myc-Parkin expression inhibited the migration (**a**) and invasion (**b**) of different human breast cells as determined by transwell assays. Left panels of **a**, **b**: representative images of migrating (**a**) or invading (**b**) MDA-MB231 cells transduced with control (Con) or Myc-Parkin vectors. Scale bars: 200 μm. Right panels of **a**, **b**: quantification of average number of migrating or invading cells per field. **c** Knockdown of endogenous Parkin by shRNA vectors promoted the migration and invasion of different human breast cells. **d** *Parkin*−/− MEFs displayed enhanced abilities of migration and invasion compared with *Parkin* + / + MEFs. Left panel: representative images of migrating or invading cells. Scale bars: 500 μm; Right panel: quantification of average number of migrating or invading cells per field. **e** Knockdown of HIF-1α largely abolished the promoting effect of Parkin knockdown on migration (upper panel) and invasion (lower panel) of cells as measured by transwell assays. Cells with HIF-1α knockdown were further transduced with control or Parkin shRNA vectors for transwell assays. **f** Expression of K477R HA-rHIF-1α largely abolished the inhibitory effect of Myc-Parkin on cell migration (upper panel) and invasion (lower panel) in MCF7 and MDA-MB231 cells. Endogenous HIF-1α in cells was replaced with WT or K477R HA-rHIF-1α (shown in Fig. 5g), and cells were then transduced with Myc-Parkin for transwell assays. **g** C431A, T173A, T240M and P294S mutations of *Parkin* compromised the inhibitory effects of Parkin on cell migration (left panel) and invasion (right panel). In **a**–**g**, the data present mean ± SD (*n* = 6). #: *P* < 0.05; *P < 0.01; **P < 0.001; two-tailed Student’s *t* test

HIF-1α plays a critical role in cancer metastasis^21, 49^. Our results show that Parkin ubiquitinates and degrades HIF-1α, suggesting that Parkin may play a critical role in inhibiting cancer metastasis through its downregulation of HIF-1α. Therefore, we investigated the effects of Parkin on the migration and invasion of various breast cancer cells and normal breast MCF10A cells using transwell assays. Cells were seeded into the upper chamber containing serum-free medium without or with matrigel for migration and invasion assays, respectively. Serum-free medium was used in the upper chamber to minimize the effect of Parkin on cell proliferation in transwell assays. Compared with cells transduced with control vectors, cells transduced with the Myc-Parkin vector showed a significantly reduced ability to migrate and invade (Fig. 6a, b). Furthermore, knockdown of Parkin significantly promoted migration and invasion of various cells (Fig. 6c). Similarly, *Parkin*−*/*− MEFs exhibited a significantly higher ability to migrate and invade than *Parkin + / + *MEFs (Fig. 6d). The inhibitory effect of Parkin on migration of breast cancer cells was further confirmed by scratch assays (Supplementary Fig. 8a, b).

We then investigated whether Parkin inhibits migration and invasion of human breast cancer cells through its downregulation of HIF-1α. Whereas knockdown of HIF-1α reduced migration and invasion of various breast cancer and MCF10A cells, knockdown of Parkin had a significantly less pronounced promoting effect on migration and invasion in cells with HIF-1α knockdown (Fig. 6e). To confirm this result, endogenous HIF-1α in MCF7 and MDA-MB231 cells was replaced with WT or K477R HA-rHIF-1α (Fig. 5g) and used for transwell assays (Fig. 6f). Whereas Myc-Parkin expression significantly inhibited migration and invasion of cells expressing WT HA-rHIF-1α, Myc-Parkin expression did not clearly affect migration or invasion of cells expressing K477R HA-rHIF-1α (Fig. 6f). Notably, C431A, T173A, T240M and P294S mutations of *Parkin* that inhibit Parkin’s E3 ligase activity for HIF-1α (Fig. 4d, h) significantly impaired the ability of Parkin to inhibit migration and invasion of breast cancer cells (Fig. 6g). Collectively, these results indicate that Parkin inhibits migration and invasion of breast cancer cells, and the negative regulation of HIF-1α contributes to this function of Parkin.

### Parkin inhibits cancer metastasis through HIF-1α regulation

**Fig. 7:**
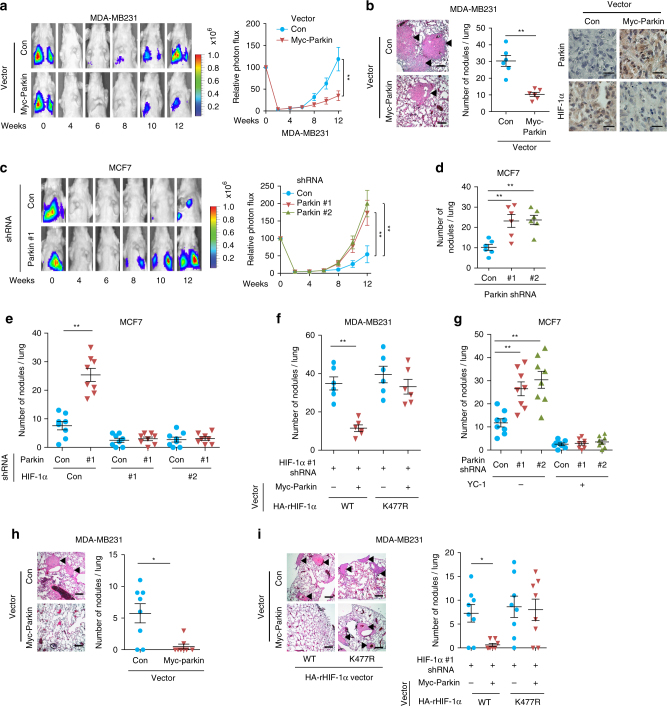
Parkin inhibits lung metastasis of human breast cancer cells in vivo. **a**, **b** Myc-Parkin expression inhibited lung metastasis of breast cancer cells injected via the tail vein. MDA-MB231 cells with Myc-Parkin expression were transduced with lentiviral vectors expressing luciferase for tail vein injections. In **a**, left panel: representative bioluminescent images at indicated time points; right panel: normalized photon flux of lung metastases. In **b**, left panel: representative H&E-stained lung sections from mice at 12 weeks after injections. Arrows indicate metastatic nodules. Scale bar: 200 μm. Middle panel: quantification of lung metastatic nodules. Right panel: IHC staining of Parkin and HIF-1α in lung metastases. Scale bar: 20 μm. **c**, **d** Knockdown of Parkin promoted lung metastasis of MCF7 cells injected via the tail vein. **e** HIF-1α knockdown largely abolished the promoting effects of Parkin knockdown on lung metastasis of MCF7 cells after tail vein injections. Two different Parkin shRNA vectors were used and similar results were observed. **f** K477R HA-rHIF-1α expression reduced the inhibitory effect of Myc-Parkin expression on lung metastasis of MDA-MB231 cells injected via the tail vein. Endogenous HIF-1α in cells was replaced with WT or K477R HA-rHIF-1α. **g** The HIF-1α inhibitor YC-1 largely abolished the promoting effect of Parkin knockdown on lung metastasis. Mice were treated with YC-1 (30 mg kg^−1^ per day; i.p.) for 5 days after the tail vein injection of MCF7 cells. **h** Myc-Parkin expression inhibited lung metastasis of MDA-MB231 cells implanted into mammary fat pads of mice. **i** K477R HA-rHIF-1α expression reduced the inhibitory effect of Myc-Parkin on lung metastasis of MDA-MB231 cells implanted into mammary fat pads. In **h**, **i**, primary tumors were surgically removed when they reached a volume of ~200 mm^3^. Left panels in **h**, **i**: representative H&E-stained lung sections at 8 weeks after primary tumor removal. Right panels in **h**, **i**: quantification of lung metastatic nodules at 8 weeks after primary tumor removal. *n* = 6 mice per group in **a**–**d**, **f**; *n* = 8 mice per group in **e**, **g**–**i**. The data present mean ± S.D. *P < 0.01; **P < 0.001; ANOVA followed by two-tailed Student’s *t* tests in **a**, **c**, and two-tailed Student’s *t* tests in the remaining panels

The lung is one of most common metastatic sites for breast cancer^50^. Therefore, we investigated the effect of Parkin on lung metastasis in vivo by employing both the tail vein injection and mammary fat pad implantation of breast cancer cells in mice. First, MDA-MB231 cells with Myc-Parkin expression and their control cells were transduced with luciferase-expressing lentiviral vectors and injected into female BALB/c athymic nude mice via the tail vein. The metastasis of cells to the lung was monitored by in vivo bioluminescence imaging. Bioluminescence imaging results showed that Myc-Parkin expression significantly inhibited lung metastasis of MDA-MB231 cells (Fig. 7a), which was confirmed by histological analysis of the lung (Fig. 7b). Furthermore, Myc-Parkin expression decreased HIF-1α protein levels in the metastases as verified by the IHC staining (Fig. 7b, right panel). We then investigated whether knockdown of endogenous Parkin in cells promotes lung metastasis using MCF7 cells that express relatively high endogenous Parkin compared with other breast cancer cell lines (Supplementary Fig. 1b). Parkin was knocked down by 2 different shRNA vectors in MCF7 cells, and cells were employed for lung metastasis analysis in mice via tail vein injections. Parkin knockdown significantly promoted lung metastasis of MCF7 cells as analyzed by in vivo imaging and histological analysis, respectively (Fig. 7c, d).

We then investigated whether negative regulation of HIF-1α mediates Parkin’s function in suppressing lung metastasis of breast cancer cells in vivo. To this end, endogenous HIF-1α was knocked down by 2 different shRNA vectors in MCF7 cells with or without Parkin knockdown. HIF-1α knockdown significantly reduced lung metastasis of MCF7 cells (Fig. 7e). Notably, a significantly less pronounced promoting effect of Parkin knockdown on lung metastasis was observed in MCF7 cells with HIF-1α knockdown (Fig. 7e). To further test this hypothesis, MDA-MB231 cells in which endogenous HIF-1α was replaced with WT or K477R HA-rHIF-1α as described in Fig. 5g were employed for tail vein injections. Myc-Parkin expression significantly inhibited lung metastasis of MDA-MB231 cells expressing WT HA-rHIF-1α, but did not clearly inhibit lung metastasis of MDA-MB231 cells expressing K477R HA-rHIF-1α (Fig. 7f). YC-1 is a widely used small-molecule HIF-1α inhibitor^51, 52^. To further test whether HIF-1α mediates the inhibitory effect of Parkin on metastasis, mice were treated daily with YC-1 (i.p.) for 5 days after tail vein injections of MCF7 cells. YC-1 treatment significantly inhibited the promoting effect of Parkin knockdown on lung metastasis of MCF7 cells (Fig. 7g).

We further investigated the effect of Parkin on lung metastasis of breast cancer cells using the mammary fat pad spontaneous metastasis model. MDA-MB231 cells with Myc-Parkin expression and their control cells were injected into the mammary fat pad for spontaneous lung metastasis (Fig. 7h). To eliminate the effect of primary tumor size on metastasis occurrence, primary tumors were surgically removed after reaching a volume of ~200 mm^3^ as described^53^. Myc-Parkin expression significantly inhibited lung metastasis of MDA-MB231 cells (Fig. 7h). To investigate whether Parkin inhibits metastasis of MDA-MB231 cells through ubiquitination and degradation of HIF-1α, we employed MDA-MB231 cells in which the endogenous HIF-1α was replaced with either WT or K477R HA-rHIF-1α (Fig. 5g) for spontaneous lung metastasis assays. Myc-Parkin expression significantly inhibited lung metastasis of cells expressing WT HA-rHIF-1α, but did not clearly inhibit lung metastasis of cells expressing K477R HA-rHIF-1α (Fig. 7i). Taken together, these results indicate that Parkin plays a critical role in suppressing breast cancer metastasis in vivo through ubiquitination and negative regulation of HIF-1α.

### Parkin expression correlates with breast cancer metastasis

**Fig. 8:**
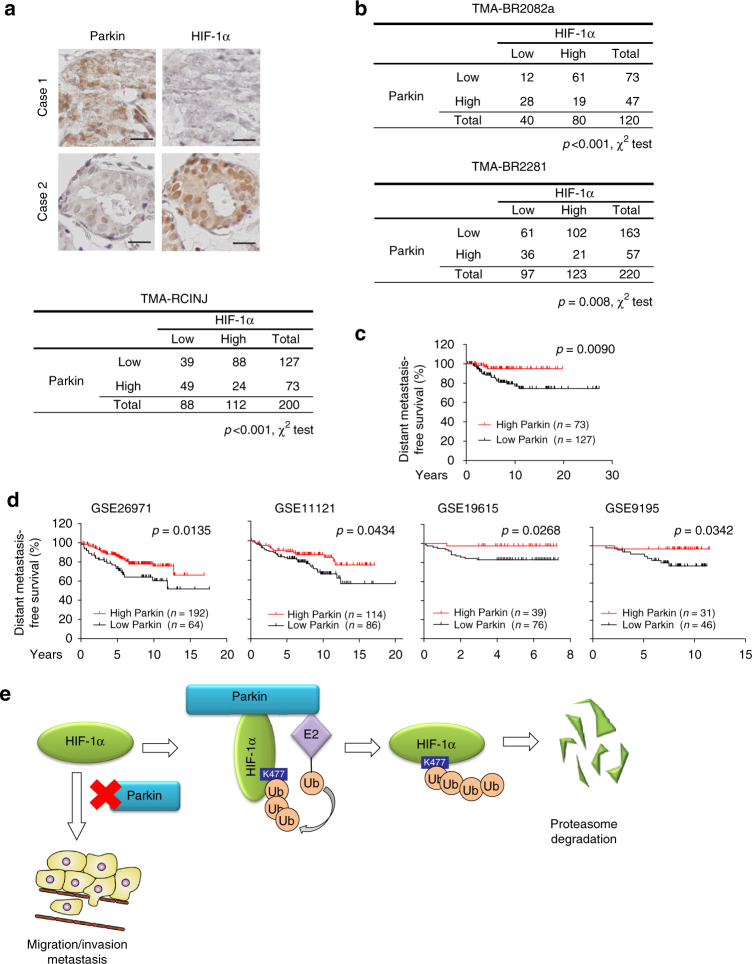
Low Parkin expression is correlated with increased HIF-1α levels and poor distant metastasis-free survival in human breast cancer. **a**, **b** Low Parkin expression was significantly correlated with increased HIF-1α levels in human breast cancer specimens analyzed by IHC staining. Upper panel in **a**: representative IHC staining images in TMA-RCINJ (*n* = 200, from the RCINJ). Scale bar: 20 µm. Lower panel in **a**: summary of IHC staining results of human breast cancer specimens in TMA-RCINJ. **b** Summary of IHC staining results in two different cohorts of human breast cancer specimens in TMAs (*n* = 120 in TMA-BR2082a; *n* = 220 in TMA-BR2281 from US Biomax). **c**, **d** Low Parkin expression was significantly associated with poor distant metastasis-free survival in human breast cancer. In **c**, low Parkin expression was significantly associated with poor distant metastasis-free survival in TMA-RCINJ. In **d**, the survival information and mRNA levels of *Parkin* were obtained from GEO databases. In **c**, **d**: Kaplan–Meier curves were drawn by GraphPad Prism software. Differences between the two survival curves were analyzed using the log-rank (Mantel–Cox) test. **e** A model depicting the suppression of cancer metastasis by Parkin-mediated HIF-1α ubiquitination and degradation

The correlation between the Parkin expression and HIF-1α levels was investigated in three different cohorts of human breast cancer specimens in three TMAs, including TMA-RCINJ from the Rutgers Cancer Institute of New Jersey (RCINJ), and TMA-BR2082a and TMA-BR2281 from US Biomax. The levels of Parkin and HIF-1α proteins were determined by IHC staining. Low Parkin expression was significantly correlated with high HIF-1α levels in these three cohorts of breast cancer (Fig. 8a, b). Furthermore, Parkin expression was not linked to any specific breast tumor subtype in terms of ER, PR or HER2 status (Supplementary Fig. 9).

To evaluate the clinical importance of Parkin in breast cancer metastasis, we analyzed the correlation between Parkin expression levels and distant metastasis-free survival. Low Parkin expression was significantly correlated with poor distant metastasis-free survival in the cohort of human breast cancer patients from the RCINJ (Fig. 8c). Similar results were obtained from four different cohorts of breast cancer patients from the publicly available Gene Expression Omnibus (GEO) database (http://www.ncbi.nlm.nih.gov/geo) (Fig. 8d). TMA-BR2082a and TMA-BR2281 were not used for analysis due to lack of clinical outcome information. These results demonstrate that Parkin expression levels are inversely correlated with HIF-1α levels and metastasis in breast cancer. In summary, our results demonstrate that Parkin is an important E3 ubiquitin ligase for HIF-1α, which inhibits breast cancer metastasis through ubiquitination and degradation of HIF-1α (Fig. 8e).

## Discussion

A growing body of evidence has suggested that Parkin is a tumor suppressor. For instance, Parkin knockdown promotes the proliferation of cancer cells both in vitro and in vivo, whereas ectopic expression of Parkin inhibits their proliferation^16, 54^. In mouse models, *Parkin*−/− mice are more susceptible to IR-induced tumorigenesis than *Parkin* + / + mice^31^, and *Parkin* + /−, *APC* min/ + mice showed increased development of intestinal adenomas than *Parkin* + / + , *APC* min/ + mice^13^. Parkin expression is frequently downregulated in cancer, including breast cancer^9, 14–16^, which was also confirmed in this study (Fig. 1a, b). In addition to loss of heterozygosity and copy number loss^9, 13^, promoter hypermethylation has been reported as a mechanism for Parkin downregulation in certain types of cancer, such as colorectal cancer^13^. Based on analysis of data from cBioportal, the mutation rate of *Parkin* is relatively low in different types of cancers, suggesting that *Parkin* mutations are not a major mechanism contributing to frequent Parkin downregulation in cancer^12^.

Currently, the mechanism underlying Parkin’s function in tumor suppression is not well-defined. The substrates of Parkin involved in tumorigenesis remain largely unknown. In this study, we identified HIF-1α as a substrate of Parkin involved in tumorigenesis. A recent study reported that Parkin expression reduced HIF-1α protein levels in cultured glioblastoma cells, however, its mechanism remains unknown^25^. Based on our results in this study, the ubiquitination of HIF-1α could be an important mechanism whereby Parkin downregulates HIF-1α in glioblastoma cells, which can be tested in future. Considering the role of Parkin in downregulation of HIF-1α, our results suggest that the frequent Parkin downregulation in breast cancer contributes to HIF-1α overexpression in cancer. Indeed, our results showed that low Parkin expression is significantly associated with high HIF-1α expression in breast cancer (Fig. 8a, b). HIF-1α plays a critical role in cancer metastasis^20, 21, 49^. Our results in this study demonstrate that Parkin inhibits migration, invasion and metastasis of breast cancer cells through its ubiquitination and degradation of HIF-1α. Furthermore, Parkin expression is significantly correlated with the poor distant metastasis-free survival in breast cancer (Fig. 8c, d). Thus, our results provide evidence that Parkin inhibits breast cancer metastasis through downregulation of HIF-1α. These results also suggest that targeting HIF-1α could be a feasible therapeutic strategy for breast cancer with Parkin downregulation.

HIF-1α is a key regulator of a broad range of cellular processes in addition to metastasis^20, 21^. HIF-1α transcriptionally regulates many downstream target genes which are involved in cell proliferation, survival, metabolic adaptation, angiogenesis and metastasis, and thus plays an important role in different steps of cancer progression^20, 21^. In this study, we examined the effect of Parkin on the expression of three well-known HIF-1α target genes involved in metastasis and/or angiogenesis, including *VEGFA, CXCR4* and *LOX*, and found that Parkin repressed their expression in a HIF-1α-dependent manner in different breast cell lines (Fig. 3c, d; Supplementary Fig. 4e, f). Currently, it remains unclear: 1) which downstream genes of HIF-1α in addition to *VEGFA, CXCR4* and *LOX* are regulated by Parkin to mediate Parkin’s function in suppression of metastasis of breast cancer cells; 2) whether Parkin can regulate different steps of cancer progression in addition to metastasis, such as cell survival, metabolic reprogramming and angiogenesis, through regulation of HIF-1α and its downstream genes; 3) whether Parkin can regulate HIF-1α signaling in other types of cancer in addition to breast cancer. It will be of interest to systemically investigate the impact of Parkin on HIF-1α levels and expression of HIF-1α downstream genes in different types of tissues and cancers. These questions should be addressed by future experiments, which could shed further light on the role and mechanism of Parkin in tumor suppression.

VHL plays an important role in the regulation of HIF-1α, which has been extensively studied^20, 21^. Accumulating evidence has shown that the VHL-independent mechanism also contributes to HIF-1α regulation^22, 23^, which is not well-understood. In this study, we found that Parkin downregulated HIF-1α protein levels in VHL-deficient RCC4 cells and MCF7 cells with VHL knockdown (Fig. 2h–j). Furthermore, Parkin downregulated HIF-1α under both normaxic and hypoxic conditions (Fig. 2e–g). While K532, K538 and K547 of HIF-1α are the major ubiquitination sites for VHL^47, 48^, K477 of HIF-1α was identified as the major ubiquitination site for Parkin in this study (Fig. 5c, d). These results indicate that Parkin can ubiquitinate HIF-1α in VHL and hypoxia-independent manners. Thus, Parkin provides an additional layer of regulation for HIF-1α in cells.

In summary, our study identified Parkin as an E3 ubiquitin ligase for HIF-1α. Through negative regulation of HIF-1α, Parkin inhibits cancer metastasis, which is an important mechanism for Parkin in tumor suppression. These results also reveal an additional mechanism for HIF-1α regulation in cells, and suggest that the frequent Parkin downregulation in breast cancer contributes to the increased HIF-1α expression in cancer.

## Methods

### Cell culture and vectors

MCF7, MDA-MB231, ZR-75-1, SK-BR3, T47D and MCF10A cells were obtained from ATCC. RCC4 and RCC4/VHL cells were generous gifts from Dr. William G. Kaelin (Harvard University). All cell lines were authenticated by short tandem repeat profiling. Cells were regularly tested for mycoplasma using Lookout Mycoplasma PCR detection kit (MP0035, Sigma) and only used when negative. The retroviral vectors expressing Myc-tagged Parkin (pLPCX-Myc-Parkin), HA-tagged HIF-1α (pLHCX-HA-HIF-1α), their deletion mutants and Flag-tagged VHL (pLPCX-VHL-Flag) were constructed by PCR amplification. pLPCX-Myc-Parkin vectors with C431A, T173A, T240M or P294S mutations, pLHCX-HA-HIF-1α vectors with different lysine residue mutations, and the WT and K477R pLHCX-HA-HIF-1α vectors resistant to HIF-1α shRNA #1 were constructed by site-directed mutagenesis using QuikChange II XL Site-Directed Mutagenesis Kit (Agilent Technologies). Detailed information on the primer sequences for site-directed mutagenesis is listed in Supplementary Table 2. Two lentiviral shRNA vectors against Parkin (#1: V3LHS_327550 and #2: V3LHS_327555), against HIF-1α (#1: V3LHS_374856, and #2: V3LHS_374854) and control shRNA vectors were obtained from Open Biosystems (Huntsville, AL). The shRNA vectors against VHL were constructed by inserting the following sequences for human VHL siRNA into the GIPZ lentiviral shRNA vector^55^. For VHL #1: 5′-AAGCCTGAGAATTACAGGAGA-3′; for VHL #2: 5′-ACACAGGAGCGCATTGCACAT-3′. Two PINK1 siRNAs were purchased from Ambion (siRNA-1: #1294; siRNA-2: #1199).

### LC-MS/MS analysis

To identify potential Parkin-binding proteins, MCF7 cells transduced with pLPCX-Myc-Parkin and MCF7 cells transduced with the empty vector were treated with MG132 (10 μM) for 8 h before being collected for assays. Myc-Parkin was pulled down by co-IP using anti-Myc beads and eluted with the Myc peptide. The elutes were then used for a second round of co-IP using an anti-Parkin antibody. To identify the ubiquitination site(s) of HIF-1α for Parkin, MCF7 cells co-transfected with vectors expressing Myc-Parkin, HA-HIF-1α and His-Ub, respectively, were treated with MG132 (10 μM) for 8 h before being harvested for assays. HA-HIF-1α was pulled down by co-IP with an anti-HA antibody and was used for LC-MS/MS analysis. LC-MS/MS analysis was performed at the Biological Mass Spectrometry facility of Rutgers University.

### Co-IP assays

Co-IP assays were performed according to standard protocols^55, 56^. For the co-IP of Myc-Parkin and HA-HIF-1α proteins, anti-Myc (9E10, Santa Cruz) and anti-HA (A2095, Sigma) agarose beads (20 µl) were used to pull down Myc-Parkin and HA-HIF-1α, respectively. For the co-IP of endogenous Parkin and HIF-1α, the anti-Parkin antibody (#4211, Cell signaling, 5 µg) and anti-HIF-1α antibody (sc-10790, Santa Cruz, 5 µg) were used, respectively. The mouse purified IgG was used as a negative control.

### Western-blot assays

Standard western-blot assays were used to analyze protein expression in cells. The following antibodies were used for assays: anti-Flag-M2 (F1804, Sigma, 1:10000), anti-β-Actin (A5441, Sigma, 1:10000), anti-Myc (9E10, Santa Cruz, 1:1000), anti-HA (#2367, Cell Signaling, 1:1000), anti-Parkin (4211, Cell Signaling, 1:500), anti-HIF-1α (ab51608, Abcam, 1:500), anti-VHL (#2738, Cell Signaling, 1:500), anti-ubiquitin antibody (sc-8017, Santa Cruz,1:2000), anti-PINK1 (ab23707, Abcam, 1:1000), anti-GST antibody (sc-138, Santa Cruz, 1:5000), and anti-His antibody (sc-803, Santa Cruz, 1:1000). The band intensity was quantified using Image J software (NIH, Bethesda). Uncropped scans of western blots presented in the main figures are provided in Supplementary Figs. 10-15.

### Protein purification and in vitro pull-down assays

Human Parkin cDNA was cloned into the GST-fusion vector pGEX-4T-1, and human HIF-1α and CDCrel-1 were cloned into the His-Trx-fusion vector pET-32a. *E. coli* (BL21 DE3 strain) transformed with various GST- or His-tagged constructs were induced for 16 h at 16 °C with 0.4 mM IPTG to express GST or His fusion proteins. To prepare GST-fusion proteins, cells were lysed by sonication in the following buffer: 25 mM Tris-HCl (PH 7.5), 100 mM NaCl, 1 mM DTT, 0.5% Triton-X-100, 10% glycerol, and protease inhibitors. GST fusions were affinity purified using Glutathione-Sepharose beads (Sigma) and bound proteins were eluted with GSH elution Buffer (20 mM reduced glutathione, 120 mM NaCl, 2 mM DTT, 20 mM Tris-HCl, PH 7.5)^41, 57^. To prepare His-tagged proteins, cells were lysed by sonication in the following buffer: 20 mM Tris-HCl, PH 7.5, 400 mM NaCl, 10 mM imidazole, 0.1% Triton X-100, 10% glycerol, 1 mM DTT, and protease inhibitors. His-tagged proteins were affinity purified using Ni-NTA agarose (Qiagen) and bound proteins were eluted with elution buffer (20 mM Tris-HCl, PH 7.5, 200 mM NaCl, 0.1% Triton-X-100, 10% glycerol, 1 mM DTT and 250 mM imidazole)^58, 59^. Purified proteins were used for in vitro GST pull-down and in vitro His pull-down assays^41, 57–59^. In brief, equal amounts of purified GST-Parkin proteins were immobilized on Glutathione-Sepharose beads, which were then incubated with purified 200 ng of His-tagged proteins or His alone. Alternatively, equal amounts of purified His-tagged proteins were immobilized on Ni-NTA agarose, which were then incubated with 200 ng of GST-fusion proteins or GST alone. After washing, the proteins bound to the beads were analyzed by western-blot with anti-His (sc-803, Santa Cruz, 1:1000) or anti-GST (sc-138, Santa Cruz, 1:5000) antibodies, respectively.

### In vivo ubiquitination assays

For in vivo ubiquitination assays, cells were transfected with vectors, including vectors expressing Myc-Parkin, HA-HIF-1α and His-Ub, respectively, for 24 h. Cells were then treated with MG132 (10 μM) for 8 h, and the levels of HA-HIF-1α ubiquitination was determined by IP with an anti-HA antibody followed by western-blot assays with an anti-Ub antibody (sc-8017, Santa Cruz,1:2000)^55^.

### In vitro ubiquitination assays

For in vitro ubiquitination assays, reaction mixtures (50 μl) consisted of buffer (5 mM MgCl_2_, 50 mM Tris, pH 7.4, 1 mM DTT, 2 mM ATP), 0.5 µg E1 (Boston Biochem), 0.5 µg E2 (UbcH7; Boston Biochem), 5 µg Ub (Boston Biochem), 0.5 µg PINK1 (Boston Biochem), 0.4 μg purified His-Trx-HIF-1α protein and 0.5 µg of purified GST-Parkin^60^. After incubation for 3 h at 37 °C, post-reaction mixtures were used for western-blot assays with an anti-Ub antibody (sc-8017, Santa Cruz, 1:2000).

### Luciferase reporter assays

For HIF-1α luciferase activity assays, cells with ectopic Myc-Parkin expression or endogenous Parkin knockdown and their controls cells were transfected with the HIF-1α luciferase reporter vector for 12 h^36, 61^. Cells were then treated with or without hypoxia (1% O_2_) for 16 h before being harvested for assays. Luciferase reporter assays were performed using the dual luciferase assay kit (Promega). The pRL-null vector expressing *renilla* luciferase (Promega) was used as an internal control to normalize the transfection efficiency.

### Quantitative real-time PCR assays

Quantitative real-time PCR was performed with TaqMan PCR mixture (Applied Biosystems) according to standard protocols^55^. The expression of genes was normalized to the expression of the *Actin* gene.

### Cell migration and invasion assays

The transwell system (8 μM pore size, BD Biosciences) was employed for cell migration and invasion assays^56, 62^. In brief, cells in serum-free medium were seeded into the upper chambers for migration assays. For invasion assays, the upper chambers were coated with matrigel. Cells on the lower surface were fixed, stained and counted at 24–48 h after seeding. Scratch assays were also performed to examine cell migration ability^63^. Cells were scratched with 20 μl pipette tips and then cultured in serum-free medium for 24 h. Scratched wound was monitored and pictures were taken at the indicated time points. The distances between the two edges of the scratched wound were measured using Image J software.

### Lung metastasis assays

In vivo lung metastasis assays were performed by the tail vein injection and orthotopic implantation of breast cancer cells in mice, respectively. For tail vein injection, MDA-MB231 and MCF7 cells (1 × 10^6^ and 2 × 10^6^ cells, respectively) transduced with lentiviral vectors expressing luciferase were injected into 2-month-old female BALB/c nude mice via the tail vein (*n* = 6 or 8 mice per group)^56, 64^. Lung metastatic colonization was monitored once every 2 weeks using bioluminescence imaging by IVIS Spectrum in vivo imaging system (PerkinElmer), and validated at the endpoint by routine histopathological analysis. For orthotopic implantation of tumor cells, MDA-MB231 cells (5 × 10^6^ in a 50:50 mix of DMEM: Matrigel) were injected into the mammary fat pad of female mice as described previously (*n* = 8 mice per group)^65^. Primary tumors were surgically removed when they reached a volume of ~200 mm^3^ to eliminate the effect of primary tumor size on metastasis occurrence^53^. Mice were killed at 8 weeks after primary tumor removal, and lung metastasis was examined by routine histopathological analysis. All mouse experiments were approved by the University Institutional Animal Care and Use Committee.

### Human breast cancer TMAs

The TMA-RCINJ (from the RCINJ) composed of 200 primary breast tumors were processed on this Rutgers institutional review board approved study^66, 67^. Annotated de-identified clinical data were entered into a database. All patients in the study were treated with breast conserving surgery followed by radiation therapy to the intact breast. Systemic therapy was administered as clinically indicated in accordance with standard clinical practice. Distant metastases were defined as clinical evidence of distant disease based on clinical and/or radiographic findings. Two other TMAs, TMA-BR2082a and TMA-BR2281, were obtained from US Biomax. TMA-BR2082a contains 120 human breast tumor tissues and 48 non-tumor breast tissues, and TMA-BR2281 contains 220 human breast tumor tissues. TMA-BR2082a and TMA-BR2281 do not include information on clinical outcomes.

### Immunohistochemistry assays

IHC staining of human breast cancer samples in TMAs was performed using anti-Parkin (NB100-91921, Novus, 1:100) and anti-HIF-1α antibodies (sc-10790, Santa Cruz, 1:50), respectively^55, 62^. Signals in tumor cells were visually quantified using a scoring system from 0 to 9. The scores were obtained by multiplying the intensity of signals with the percentage of positive cells (signal: 0 = no signal, 1 = weak signal, 2 = intermediate signal, and 3 = strong signal; percentage: 0 = 0%, 1 ≤ 25%, 2 = 25-50%, and 3 ≥ 50%). Low and high expression were defined as scores of < 6 and ≥ 6, respectively^55, 68^.

### Statistical analysis

No statistical methods were used to predetermine sample size. For experiments, a minimum of three samples were chosen as a sample size to ensure adequate power. The experiments were not randomized. The investigators were not blinded to allocation during experiments and outcome assessment. The differences in tumor growth among groups were analyzed for statistical significance by analysis of variance (ANOVA), followed by two-tailed Student’s *t* test using GraphPad Prism software. The differences between two Kaplan–Meier survival curves were analyzed by the log-rank (Mantel–Cox) test using GraphPad Prism software. *P* values < 0.05 were considered statistically significant and set as follows: #*P* < 0.05; *P < 0.01; **P < 0.001.

### Data availability

Parkin expression and alterations data were obtained from the public TCGA portal (https://tcga-data.nci.nih.gov/tcga/) and from Cbioportal (http://www.cbioportal.org/), respectively. The relapse-free survival data and the metastasis-free survival data were obtained from Kaplan–Meier plotter (http://kmplot.com/analysis/), and from Gene Expression Omnibus (GEO) database (https://www.ncbi.nlm.nih.gov/geo/), respectively. All other remaining data are available in the article and Supplementary Files, or available from the authors upon request.

## Electronic supplementary material

Supplementary Information
