# Supplementary material for: Parkin targets HIF-1α for ubiquitination and degradation to inhibit breast tumor progression
Source: Nat Commun. 2017 Nov 28;8:1823. doi: 10.1038/s41467-017-01947-w (PMC5703960; doi:10.1038/s41467-017-01947-w)
Supplement: Supplementary file 1 — Supplementary Information [file 41467_2017_1947_MOESM1_ESM.pdf]

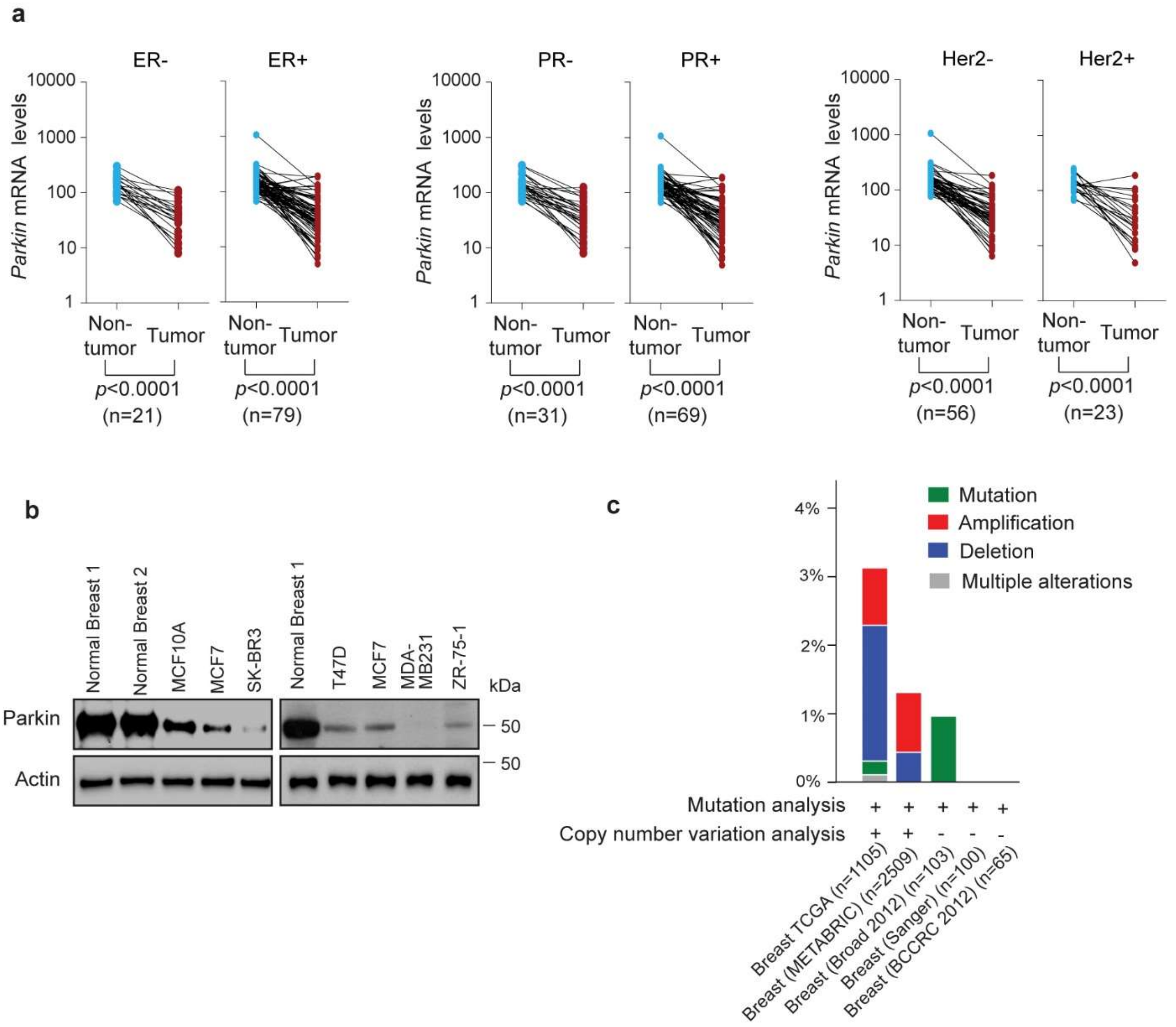

Supplementary Figure 1. *Parkin* alterations in human breast cancer, and Parkin protein levels in human breast cancer cell lines. (**a**) The downregulation of *Parkin* expression was not linked to any specific subtype of human breast cancers. The mRNA levels in breast cancer samples were compared with matched adjacent non-tumor breast tissues (n=113). Data were obtained from TCGA. (**b**) The protein levels of Parkin in normal breast tissues, MCF10A cells, and different human breast cancer cells measured by western-blot assays. (**c**) Summary graph of *Parkin* alterations in different cohorts of breast cancers from individual studies deposited in cBioPortal.

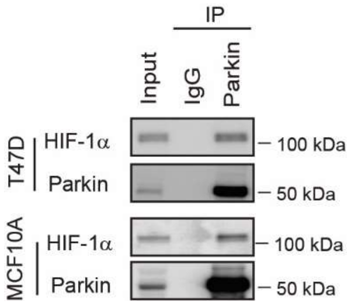

Supplementary Figure 2. Endogenous Parkin interacts with endogenous HIF-1α in T47D and MCF10A cells. Endogenous Parkin in T47D and MCF10A cells was pulled down by co-IP assays using an anti-Parkin antibody and endogenous HIF-1α was detected by western-blot assays.

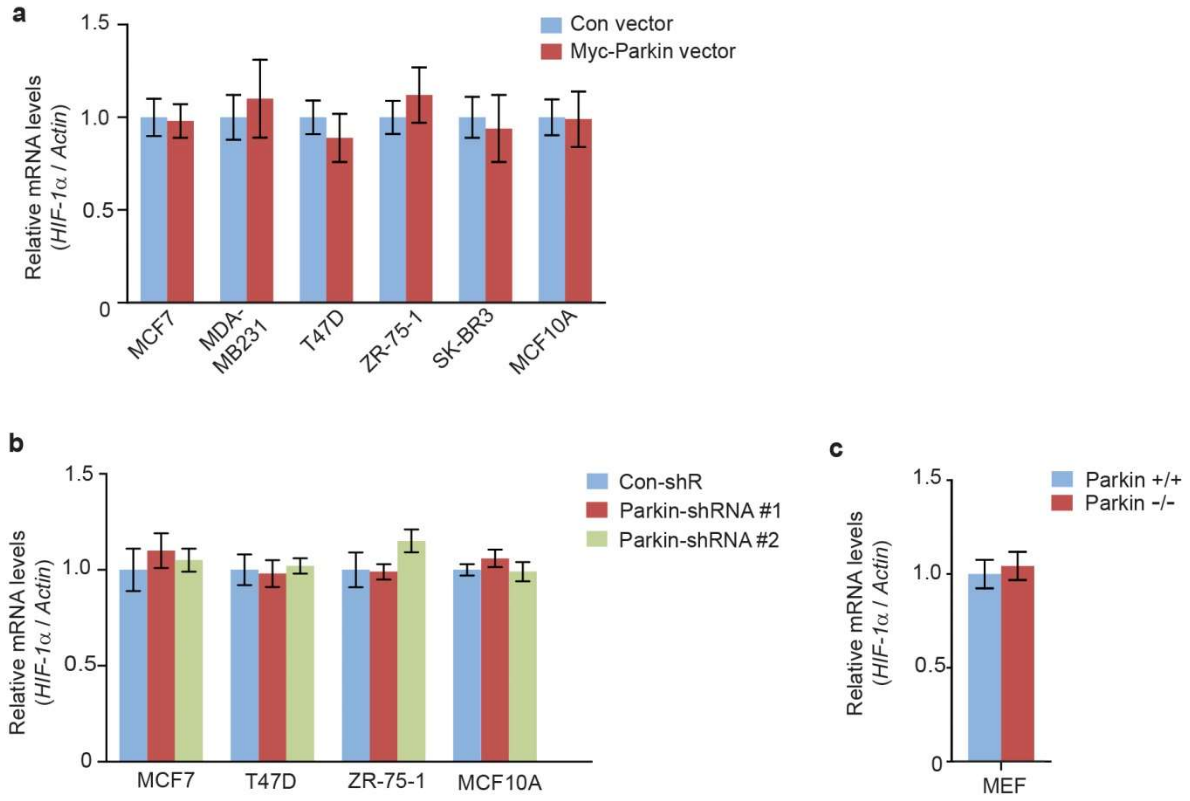

Supplementary Figure 3. Parkin expression does not affect the mRNA levels of *HIF-1α* in human breast cells. (**a**) Ectopic expression of Myc-Parkin did not affect the mRNA levels of *HIF-1α* in different human breast cells. (**b**) Knockdown of endogenous Parkin by 2 different shRNA vectors did not affect the mRNA levels of *HIF-1α* in different human breast cells. (**c**) No difference in *HIF-1α* mRNA expression levels was observed in Parkin +/+ and Parkin -/- MEFs. In (**a-c**), the mRNA levels of *HIF-1α* were measured by quantitative Taqman real-time PCR assays and normalized with *actin*. Data present mean ± S.D. (n=3).

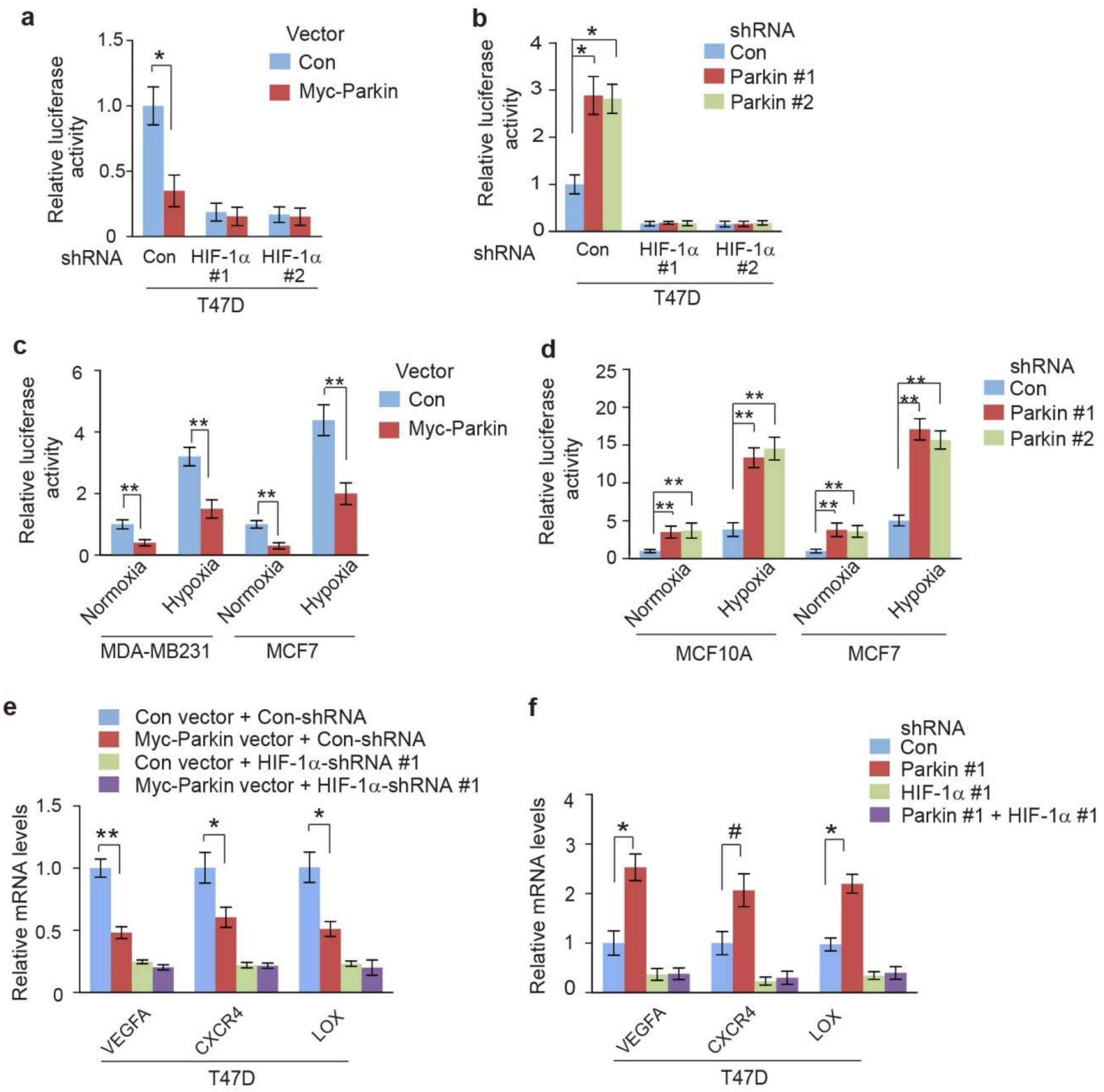

Supplementary Figure 4. Parkin negatively regulates HIF1-α transcriptional activity. (**a**) Ectopic Myc-Parkin expression inhibited HIF-1α luciferase reporter activities in T47D cells. (**b**) Knockdown of endogenous Parkin increased HIF-1α luciferase reporter activities in T47D cells. (**c**) MCF7 and MDA-MB-231 cells transfected with either control or Myc-Parkin expression vectors together with HIF-1α luciferase reporter vectors were cultured under conditions of normoxia (20% O_2) or hypoxia (1% O_2) for 12 h, and then subjected to luciferase activity assays. (**d**) MCF7 and MCF10A cells with Parkin knockdown by shRNA vectors and their control cells were cultured under conditions of normoxia or hypoxia for 12 h, and then subjected to luciferase activity assays. (**e**) Myc-Parkin expression reduced mRNA expression of HIF-1α target genes in T47D cells. (**f**) Parkin knockdown increased mRNA expression of HIF-1α target genes in T47D cells. Gene expression was measured by quantitative Taqman real-time PCR and normalized with Actin. In (**e**), HIF-1α was knocked down by 2 different shRNA vectors, and in (**f**), HIF-1α and Parkin were knocked down by 2 different shRNA vectors. Similar results were observed for 2 different HIF-1α and Parkin shRNA vectors, and only results from one shRNA vector were presented for the sake of clarity. Data present mean ± S.D. (n=3 for a,b,e,f; n=6 for c,d). **: P<0.001; *: P<0.01; #: P<0.05; two-tailed Student's t-test.

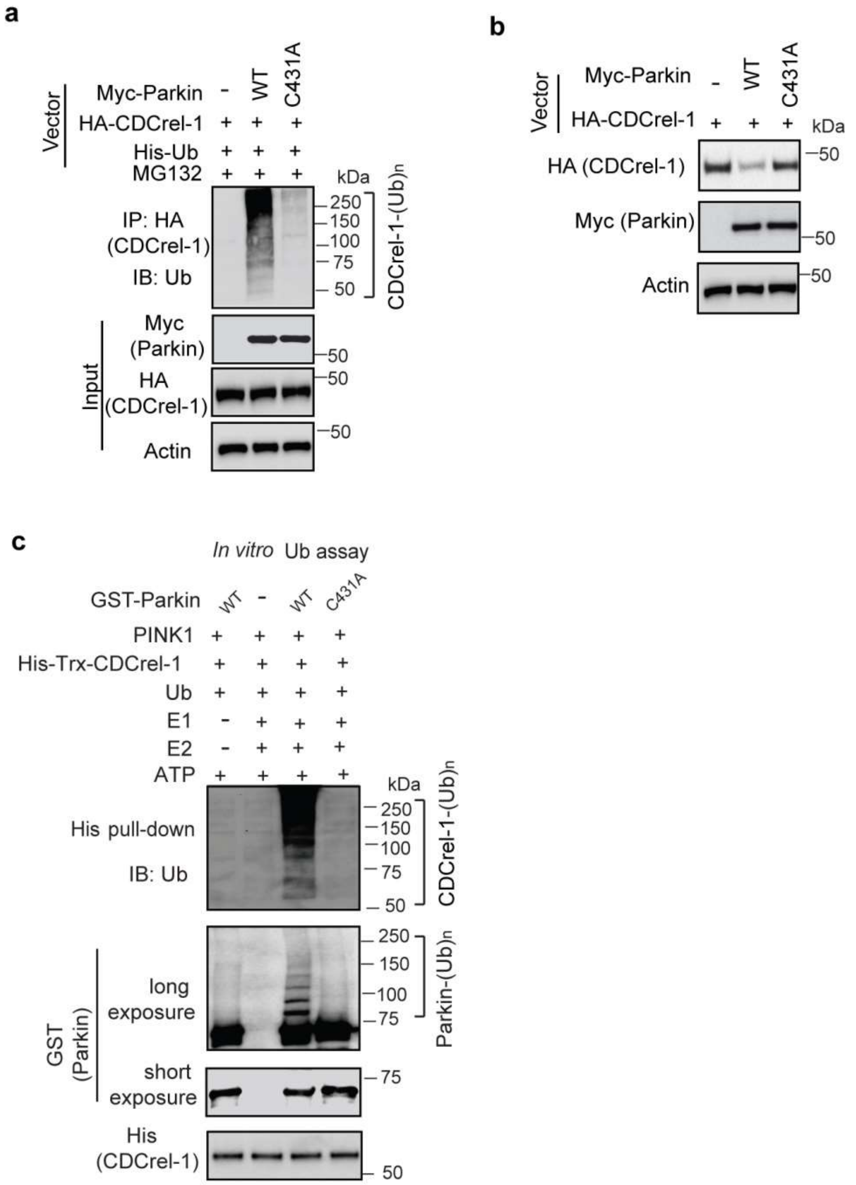

Supplementary Figure 5. Parkin promotes CDCrel-1 protein ubiquitination and degradation. (**a**) Expression of WT but not C431A Myc-Parkin promoted the ubiquitination of HA-CDCrel-1 in MCF7 cells analyzed by *in vivo* ubiquitination assays. (**b**) Expression of WT but not C431A Myc-Parkin down-regulated HA-CDCrel-1 protein levels analyzed by western-blot assays in MCF7 cells. (**c**) WT but not C431A GST-Parkin promoted CDCrel-1 ubiquitination analyzed by *in vitro* ubiquitination assays performed by incubating purified GST-Parkin and His-Trx-CDCrel-1 proteins with recombinant E1, E2, Ub and PINK1 proteins *in vitro*.

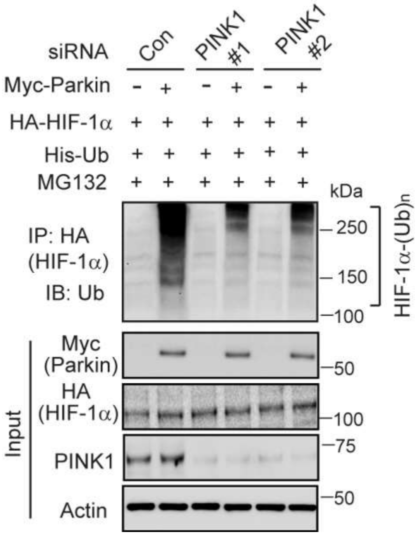

Supplementary Figure 6. Knockdown of endogenous PINK1 impaires HIF-1α ubiquitination mediated by Parkin in cells. Knockdown of endogenous PINK1 by siRNA oligos impaired the ubiquitination of HA-HIF-1α by Myc-Parkin in MCF7 cells analyzed by *in vivo* ubiquitination assays. MCF7 cells were transfected with 2 different siRNA oligos to knock down endogenous PINK1, and then transfected with Myc-Parkin, HA-HIF-1α and His-Ub expression vectors for *in vivo* ubiquitination assays.

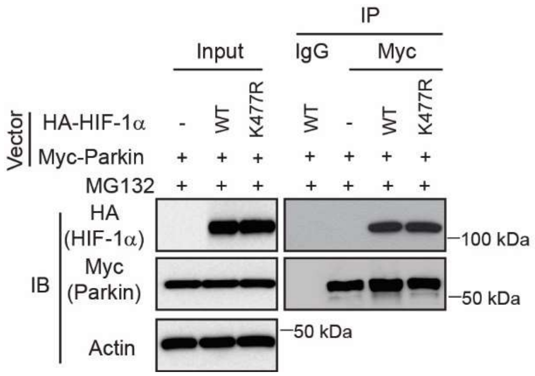

Supplementary Figure 7. K477R mutation of HIF-1α does not affect the interaction between HA-HIF-1α and Myc-Parkin in MCF7 cells analyzed by co-IP assays. Cells were co-transduced with vectors expressing WT or K477R HA-HIF-1α together with Myc-Parkin expression vectors for co-IP assays using an anti-Myc antibody.

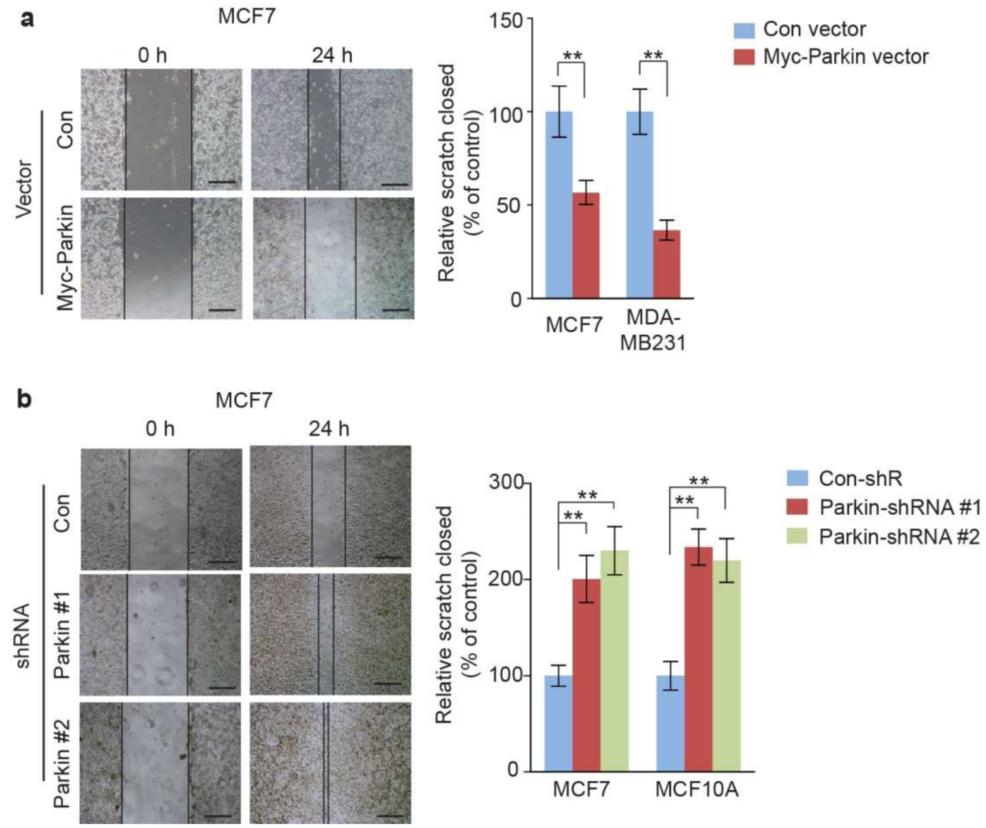

Supplementary Figure 8. Parkin inhitits migration of breast cancer cells as determined by scratch assays. (**a**) Ectopic Myc-Parkin expression in MCF7 cells inhibited cell migration as determined by scratch assays. (b) Knockdown of endogenous Parkin by shRNA vectors in MCF7 cells promoted cell migration as determined by scratch assays. Left panels in (**a**, **b**): representative images of MCF7 cells transduced with indicated vectors in scratch assays. Right panels in (**a**, **b**): quantification of scratch assays. The distances between the two edges of the scratched wounds were measured at 24 h after stratching. Data present mean ± S.D. (n = 6). **: P<0.001; two-tailed Student t-test . Scale bar: 200 μm.

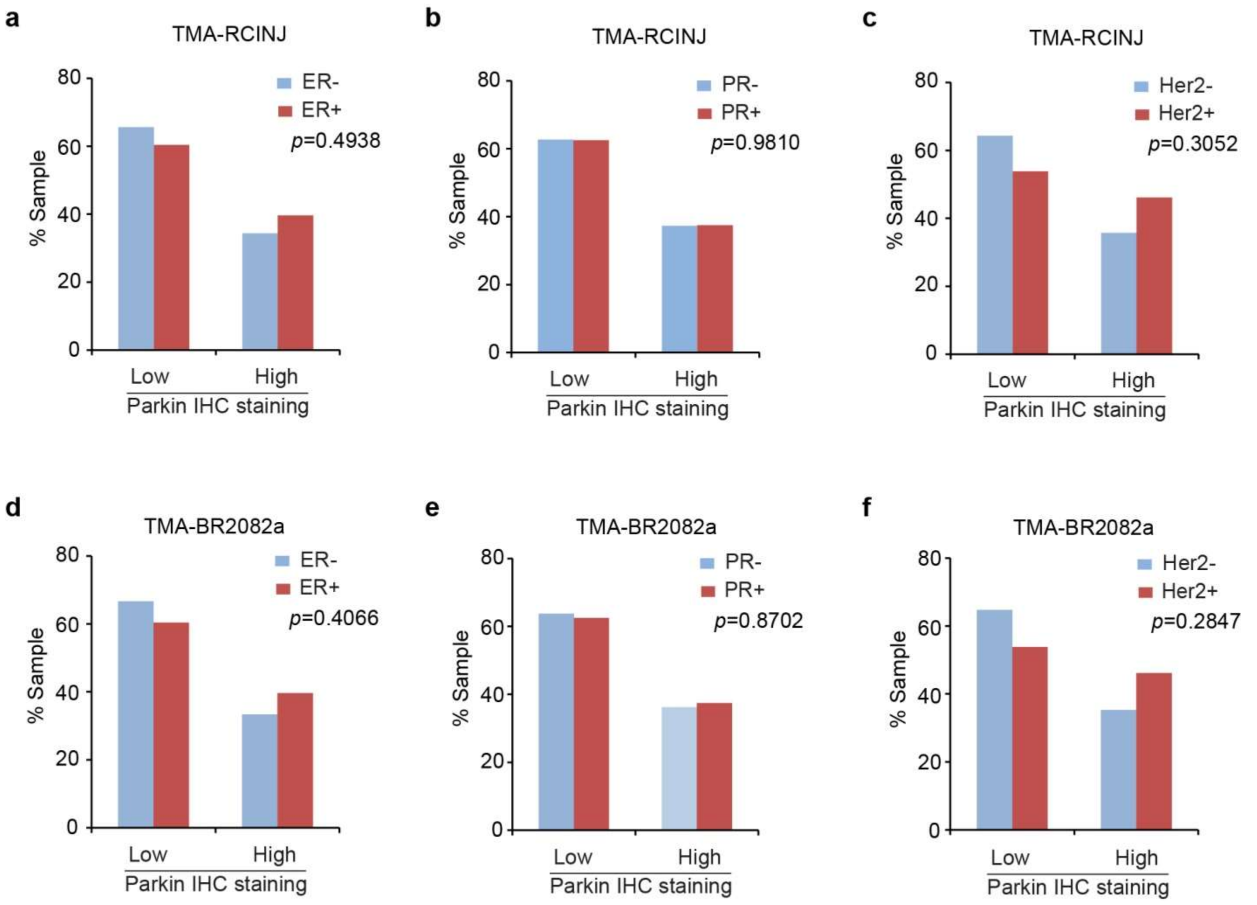

Supplementary Figure 9. Parkin expression is not linked to any specific subtype of breast cancer. Parkin protein levels in two breast cancer TMAs (TMA-RCINJ and TMA-BR2082a) were detected by IHC staining, and were compared in different cancer subtypes classified by status of ER (**a**, **d**), PR (**b**, **e**), or HER2 (**c**, **f**). Statistical studies were performed by using $\chi 2$ test. The TMA-BR2281 did not have information on status of ER, PR or HER2.

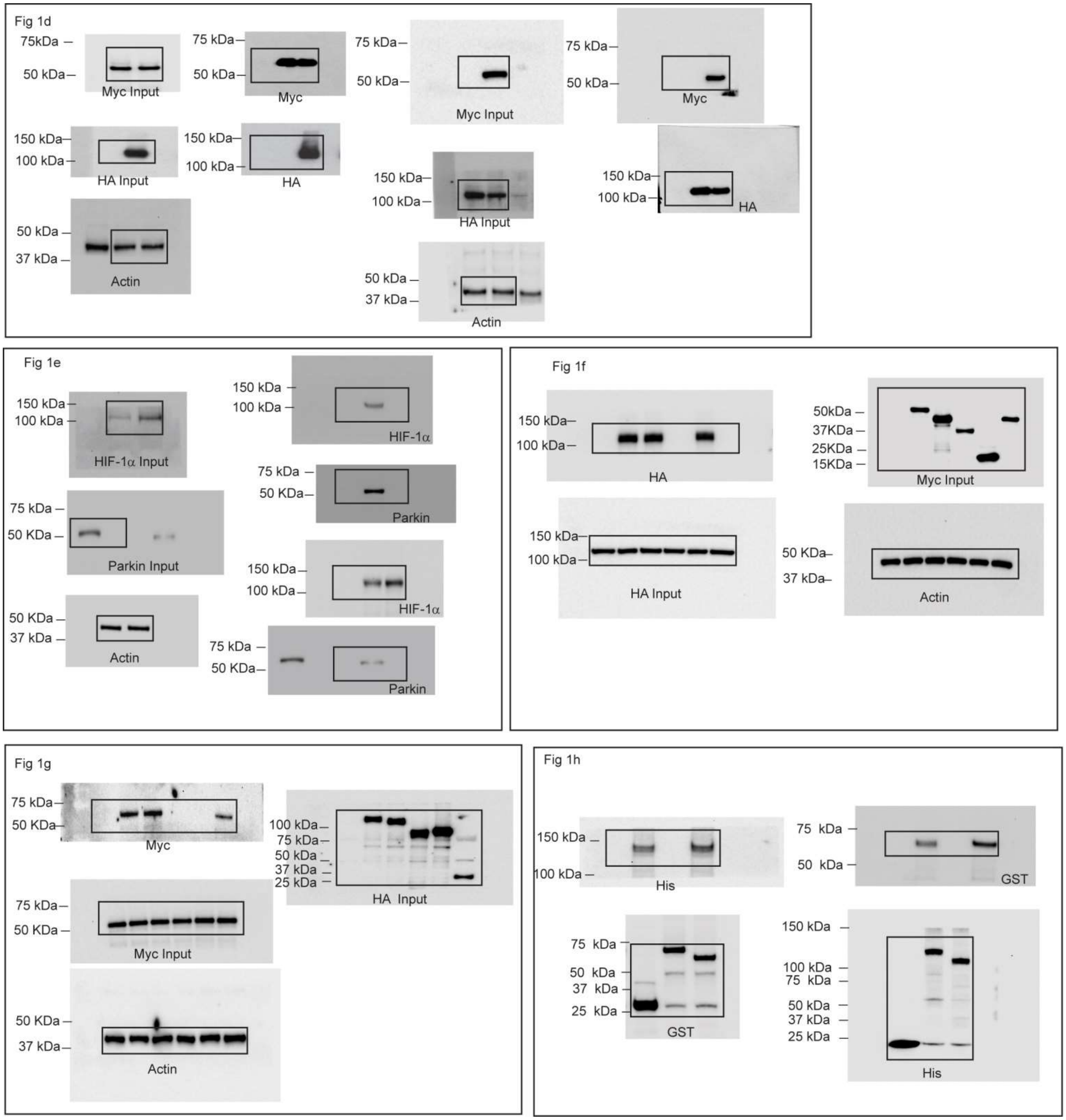

Supplementary Figure 10. Uncropped blots for Figure 1. The boxed regions are presented in the indicated figures in the manuscript.

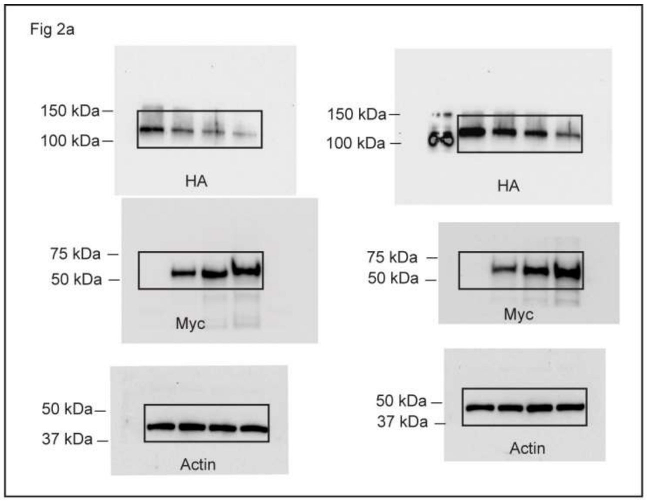

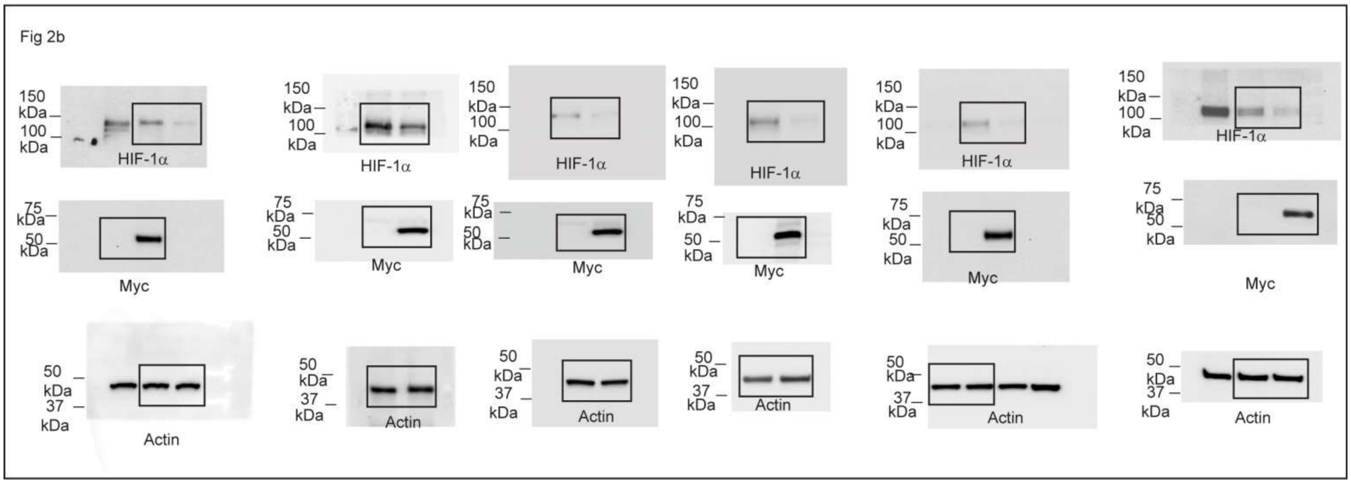

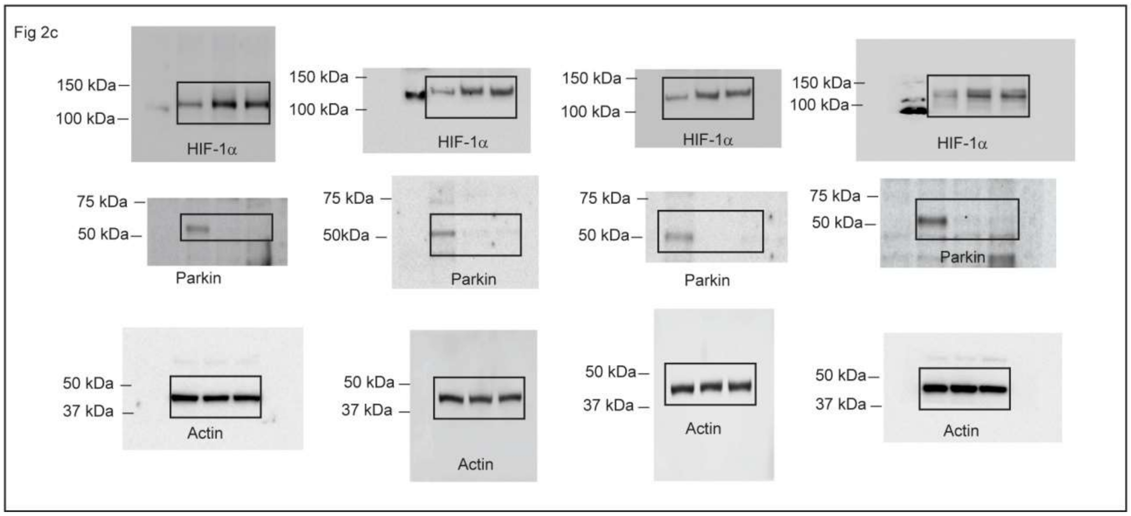

Supplementary Figure 11. Uncropped blots for Figure 2a-2c. The boxed regions are presented in the indicated figures in the manuscript.

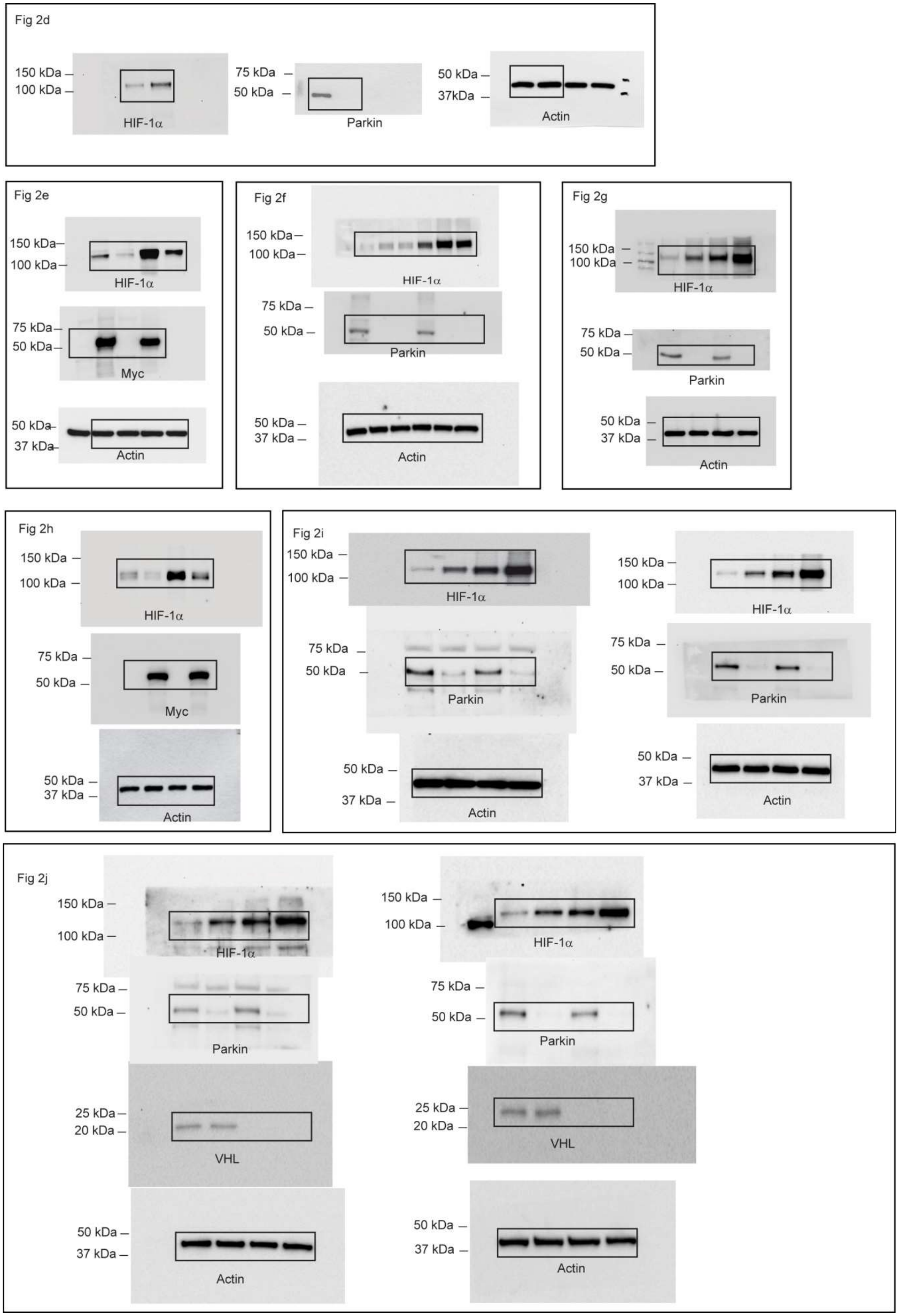

Supplementary Figure 12. Uncropped blots for Figure 2d-2j. The boxed regions are presented in the indicated figures in the manuscript.

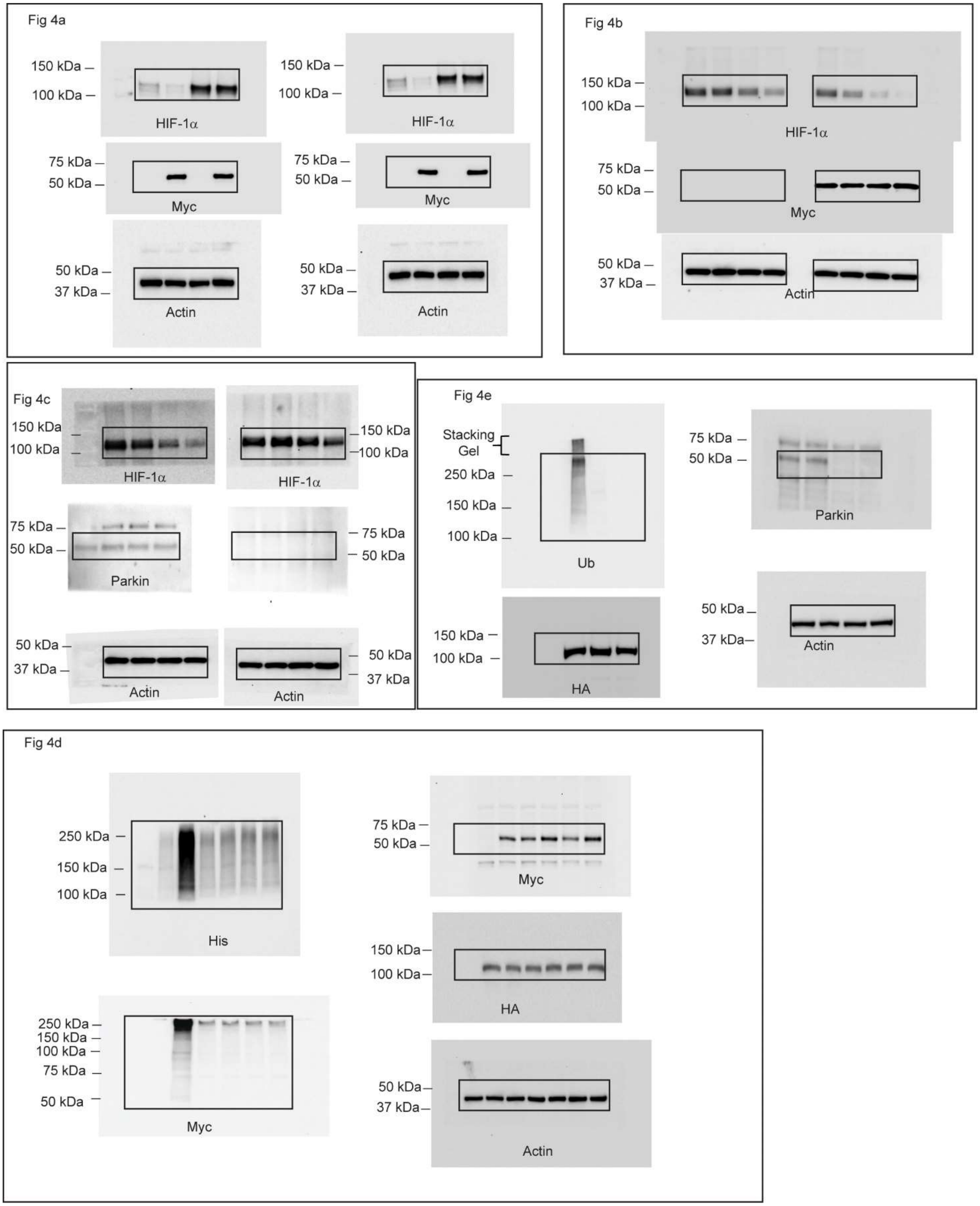

Supplementary Figure 13. Uncropped blots for Figure 4a-4e. The boxed regions are presented in the indicated figures in the manuscript.

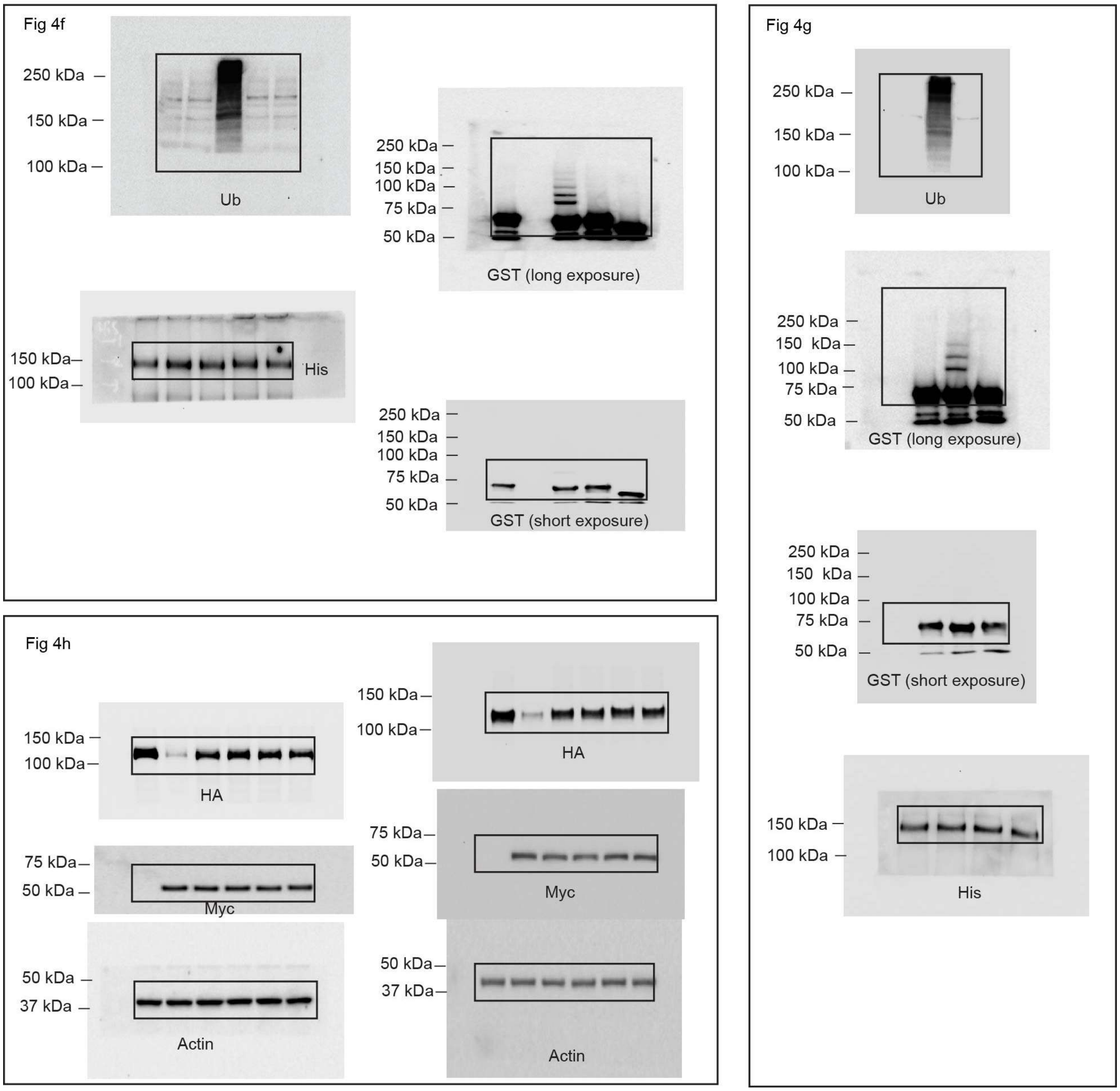

Supplementary Figure 14. Uncropped blots for Figure 4f-4h. The boxed regions are presented in the indicated figures in the manuscript.

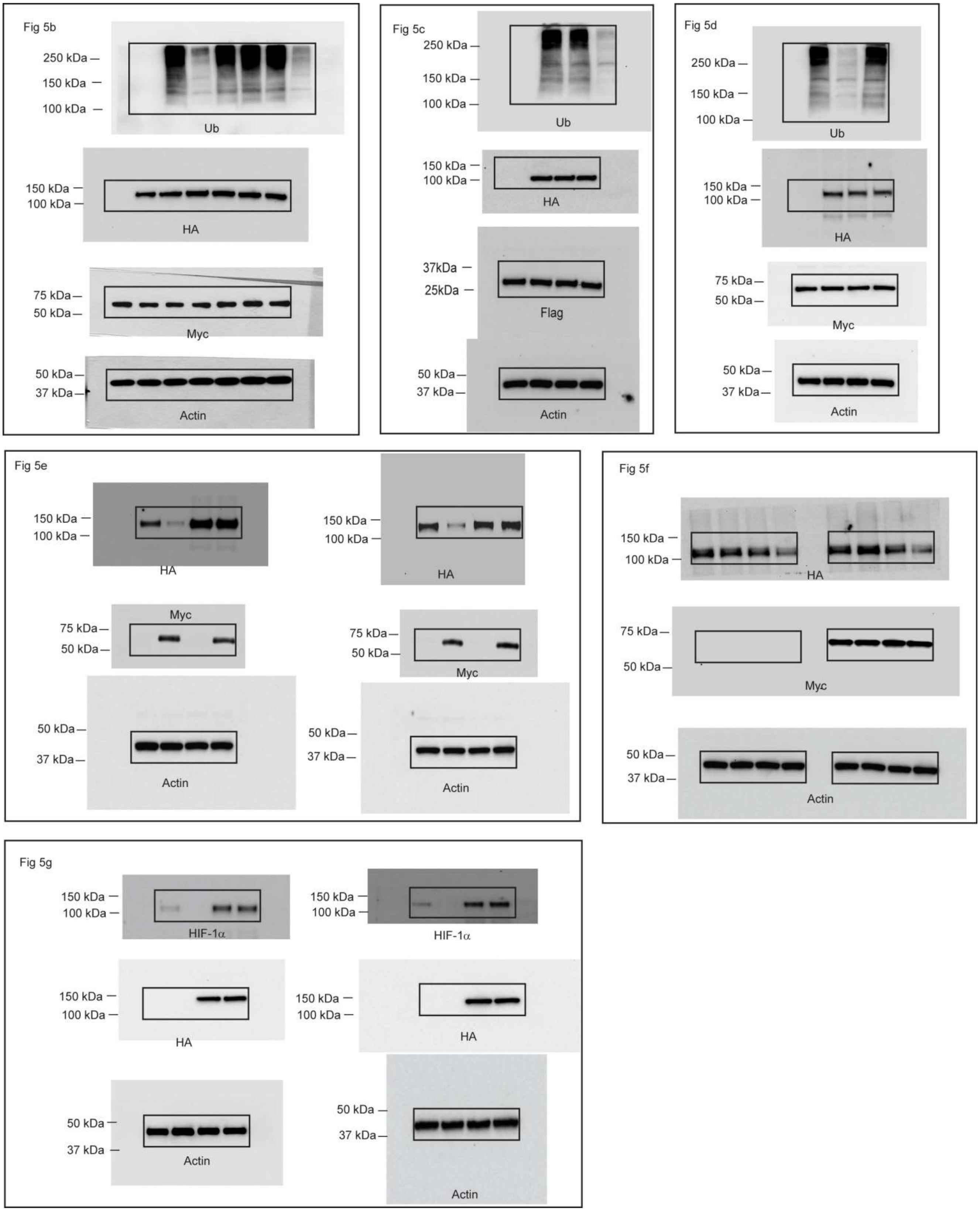

Supplementary Figure 15. Uncropped blots for Figure 5. The boxed regions are presented in the indicated figures in the manuscript.

Supplementary Table 1. The potential Parkin-interacting proteins identified by co-IP followed by LC-MS/MS assays

Potential interacting proteins for Parkin	Number of peptides	
	Control	Parkin
Laminin	0	17
HSP70	0	15
Rpn10	0	5
UBL4A	0	5
**HIF-1α**	**0**	**4**
OSGIN1	0	4
TOMM40	0	4
ABCE1	0	4
14-3-3	0	3
ATAD1	0	2
Tubulin	0	2

MCF7 cells with Myc-Parkin overexpression and control MCF7 cells were treated with MG132 before being harvested for co-IP assays. Myc-Parkin was pulled down by two sequential rounds of co-IP using anti-Myc beads and then the anti-Parkin antibody before LC-MS/MS assays.

Supplementary Table 2. Sequences of primers

Primer Name	Forward (5'-3')	Reverse (5'-3')
Parkin C431A	AGTGGAAAAAAATGGAGGCGCCATGCACATGAAGTGTCCG	CGGACACTTCATGTGCATGGCGCCTCCATTTTTTTCCACT
Parkin T173A	GCACCTGCAGGCAGGCAGCGCTCACCT	AGGTGAGCGCTGCCTGCCTGCAGGTGC
Parkin T240M	AGTCGGAACATCACTTGCATTATGTGCACAGACGT	ACGTCTGTGCACATAATGCAAGTGATGTTCCGACT
Parkin P294S	GCCTTGTGTGGCTGGCTGTTCCAACTCCTT	AAGGAGTTGGAACAGCCAGCCACACAAGGC
HIF-1α K477R	ACTCAATCAAGAAGTTGCATTAAGATTAGAACCAAATCCAGAGTCA	TGACTCTGGATTTGGTTCTAATCTTAATGCAACTTCTTG ATTGAGT
HIF-1α K547R	GCTGAAGACACAGAAGCAAGGAACCCATTTTCTACTCAG	CTGAGTAGAAAATGGGTTCCTTGCTTCTGTGTCTTCAGC
HIF-1α K538R	TGAATTCAAGTTGGAATTGGTAGAAAGACTTTTTGCTGAAGACACAG	CTGTGTCTTCAGCAAAAAGTCTTTCTACCAATTCCAACTTGAATTCA
HIF-1α K532R/ K538R	GATAGTGATATGGTCAATGAATTCAGGTTGGAATTGGTAGAAAGACTTTTT	AAAAAGTCTTTCTACCAATTCCAACCTGAATTCATTGAC CATATCACTATC
rHIF-1α	AACCGGTTGAATCTTCAGATATGAAAATGACTCA**AT**T**G**TT**T**AC**A**AAAGTTGAATCAGAAGATACAAGTAGCCTCTTTG	CAAAGAGGCTACTTGTATCTTCTGATTCAACTTT**T**GT**A**AA**C**A**AT**TGAGTCATTTTCATATCTGAAGATTCAACCGGTT
